# Identifying candidate gut microbiota indicators for Alzheimer’s disease through integrated data

**DOI:** 10.1128/msystems.01754-25

**Published:** 2026-03-25

**Authors:** Jing Wang, Hanting Liu, Hao Lai, Yining Bao, Mingwang Shen, Chao Li, Lu Ma, Ting Wu, Siyu Yang, Xinyu Du, Terence J. O'Brien, Jing Zhang, Lei Zhang

**Affiliations:** 1 Phase I clinical trial research ward, The Second Affiliated Hospital of Xi’an Jiaotong University 117799, Xi'an, Shaanxi, China; 2 China-Australia Joint Research Centre for Infectious Diseases, School of Public Health, Xi'an Jiaotong University Health Science Centre, Xi'an, Shaanxi, China; 3 Department of Medical Statistics, School of Public Health, Sun Yat-sen University 26469 , Guangzhou, Guangdong, China; 4 Key Laboratory for Disease Prevention and Control and Health Promotion of Shaanxi Province 631989 , Xi'an, Shaanxi, China; 5 Department of Epidemiology and Health Statistics, School of Public Health, Xi'an Jiaotong University Health Science Centre 599476, Xi'an, Shaanxi, China; 6 School of Public Health, Xi'an Jiaotong University Health Science Centre 599469, Xi'an, Shaanxi, China; 7 Department of Neurology, The First Affiliated Hospital of Nanjing Medical University 74734 , Nanjing, China; 8 Department of Neurology, The Second Hospital of Nanjing 531909, Nanjing, China; 9 The Department of Neuroscience, The School of Translational Medicine, Faculty of Medicine, Nursing and Health Sciences, Monash University & Alfred Health 2541, Clayton, Victoria, Australia; 10 Artificial Intelligence and Modelling in Epidemiology Program, Melbourne Sexual Health Centre, Alfred Health 198098, Carlton, Victoria, Australia; National Institutes of Health, Bethesda, Maryland, USA

**Keywords:** Alzheimer's disease, gut microbiota, candidate indicator, discriminative model, random forest

## Abstract

Alzheimer’s disease (AD) is associated with the gut microbiota, and identifying reliable gut microbiota biomarkers enhances AD diagnosis. We aim to characterize the gut microbiota in AD patients by integrating data from multiple populations and identifying key candidate gut microbiota indicators with diagnostic value for AD. Public data from studies on AD and gut microbiota were collected, including participants from AD dementia, mild cognitive impairment (MCI), and normal control (NC) groups. Microbiota composition, diversity, and network analyses were used to characterize the gut microbiota of the three groups. Differential bacterial genera identified simultaneously by seven common methods served as candidate indicators. The study included 799 AD dementia, 170 MCI, and 731 NC participants. The AD dementia group demonstrated a lower relative abundance of *Bacteroides* and *Faecalibacterium* and lower α-diversity than the MCI and NC groups (*P* < 0.05). The microbial network density in the AD dementia group was reduced by 1.5% and 1.6% compared with the NC and MCI groups, respectively. We identified 35 bacterial genera as candidate indicators for AD, including first-time reports of *RF39* and *Oligella. Faecalibacterium* was the most important candidate indicator in the overall population, *Akkermansia* in the Chinese population, *Collinsella* in the “Turkish and Kazakh” population, and *Actinomyces* in the “American and Canadian” population. Our findings contribute to the development of non-invasive biomarkers for AD diagnosis and targeted microbiota therapies and provide a valuable reference for selecting specific biomarkers for different application scenarios.

**IMPORTANCE:**

This study characterized the gut microbiota of Alzheimer's disease (AD) patients and identified candidate indicators for AD diagnosis using a large, multi-population data set. The AD dementia group consistently showed lower α-diversity and a sparser microbiota interaction network than the other groups. We identified 35 bacterial genera as candidate indicators for AD, including first-time reports of *RF39* and *Oligella*. *Faecalibacterium* was the most important candidate indicator in the overall population, *Akkermansia* in the Chinese population, *Collinsella* in the “Turkish and Kazakh” population, and *Actinomyces* in the “American and Canadian” population. These findings provide a valuable reference for selecting biomarkers for different application scenarios.

## INTRODUCTION

Alzheimer’s disease (AD) is a chronic neurodegenerative disorder primarily characterized by cognitive impairment in older adults. AD typically presents with a gradual decline in episodic memory and executive functions, affecting daily living abilities and life quality (1). The diagnostic criteria proposed by the U.S. National Institute on Aging and Alzheimer’s Association classify AD into preclinical, mild cognitive impairment (MCI), and dementia stages (2). Gustavsson et al. estimated that in 2020, 315 million people aged >50 years worldwide were in the preclinical stage, 69 million in the MCI stage, and 32 million in the dementia stage, accounting for nearly a quarter of the population in this age group (3). With the growing pace of aging worldwide, the number of individuals affected by AD is expected to rise, posing substantial disease and economic burdens on individuals, families, and the healthcare systems.

The pathogenic mechanism of AD is complex and not well understood. The β-amyloid protein cascade hypothesis (4) and the tau protein hyperphosphorylation hypothesis (5), which damage neuronal cells through the formation of β-amyloid plaques and neurofibrillary tangles, are among the prominent hypotheses explaining the etiology of AD. However, medications developed based on these hypotheses for treating patients with AD dementia have shown a failure rate as high as 99.6% during 2002–2012 (6). This indicates a significant knowledge gap between existing hypotheses and the underlying pathogenic mechanisms, which demands further exploration. The difficulty of managing AD also suggests that early diagnosis and prevention would be more effective.

The recent proposal of a microbiota-brain-gut axis has provided a new approach to understanding the interaction between gut microbiota and the central nervous system (CNS) (7). Accumulating evidence indicates that the dysregulation of the microbiota-brain-gut axis substantially contributes to the development of AD (8). This association not only offers a new avenue for understanding the pathogenesis of AD but also provides opportunities for exploring biomarkers for early AD diagnosis. To date, research on the association of AD and gut microbiota remains in its infancy, with most studies conducted on animal models (9–11). Only in 2017 did Vogt et al. conduct the first population study of AD and gut microbiota in the United States (12); since then a series of studies have been conducted in China (13–21), Kazakhstan (22), Turkey (23), the United States (24), Canada (25), and others. However, individual studies suffer limitations, such as small samples, relatively mono-specific populations, and inconsistent findings. A systematic and pooled analysis of existing evidence would be vital for understanding the gut microbiota characteristics in patients with AD and identifying candidate gut microbiota indicators that could be valuable for AD diagnosis.

In this study, we aim to investigate the gut microbiota characteristics of AD patients in MCI and clinical dementia stages by integrating data from multiple populations and identifying key candidate gut microbiota indicators with diagnostic values for these two disease stages. This understanding will provide a foundation for future research into early diagnosis, ultimately contributing to the development of non-invasive diagnostics and targeted microbiota therapies for personalized clinical management.

## RESULTS

### Characteristics of the data sets

**Fig 1:**
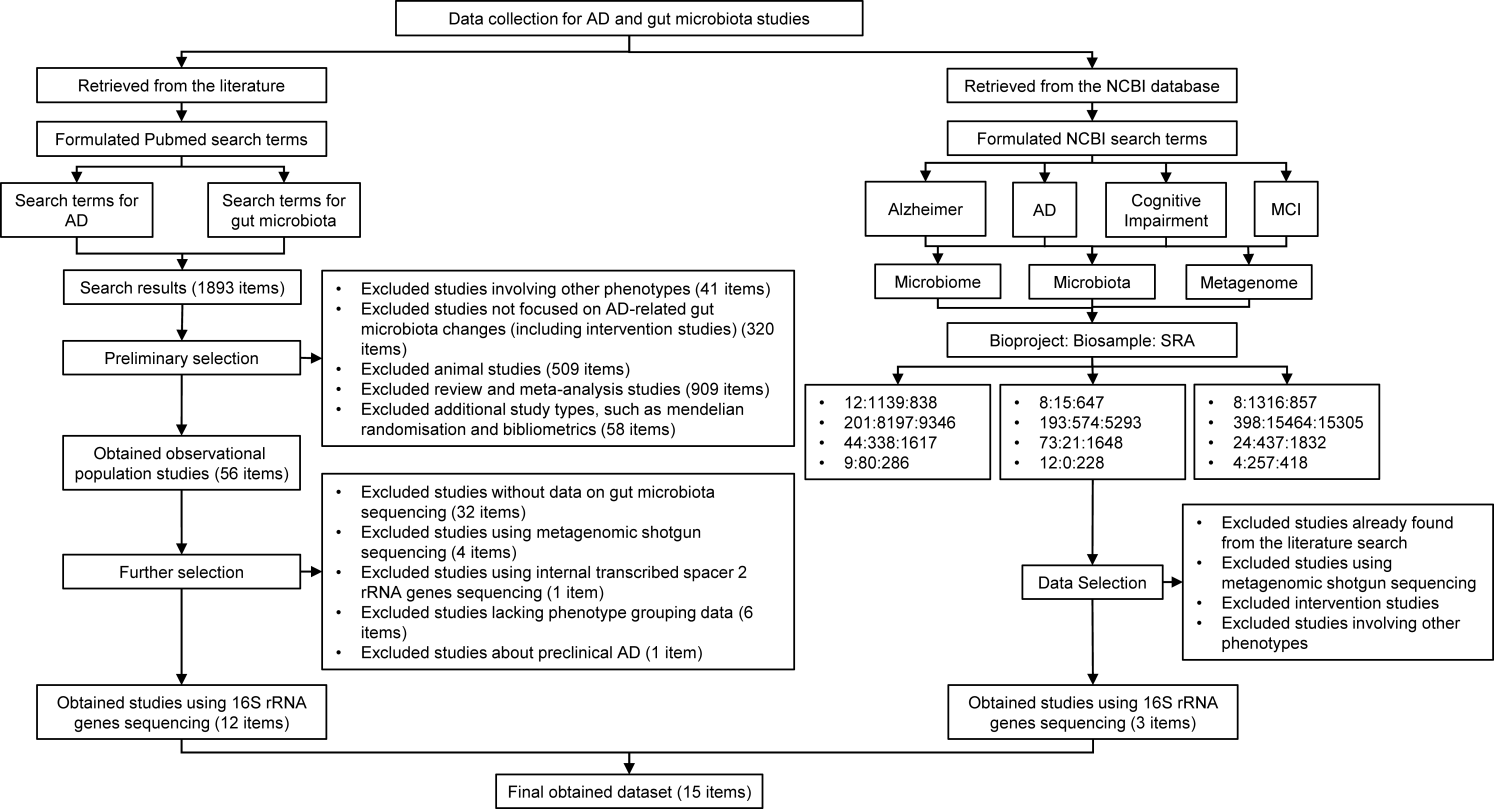
Flow chart of data collection. AD: Alzheimer’s disease; MCI: mild cognitive impairment; SRA: Sequence Read Archive.

This study collected data through two approaches (Fig. 1), including participants from AD dementia, MCI, and normal control (NC) groups. Finally, we included 1,700 participants from 15 data sets; 799 participants belonged to the AD dementia group, 170 to the MCI group, and 731 to the NC group (Table 1). After batch correction, the variance attributed to the batch factor was greatly reduced from 19.29% to 7.00% (Fig. S1). Age and sex information extracted from the original studies did not exhibit statistically significant differences between the groups. Most studies used amplification sequencing targeting the V3–V4 region to identify gut microbiota, with nine studies conducted in China, two in Kazakhstan and Turkey, and four in the United States and Canada.

**TABLE 1:** Characteristics of data sets used in the study^*a*^

Data sources	Group (*N*)	Age	Sex (% F/% M)	Sequence region	Layout	Sample geolocation
PRJNA496408 (13)	AD dementia (33)	74.9 ± 11.4	42.4/57.6	V3–V4	Paired	Hangzhou, China
	MCI (32)	70.5 ± 11.0	56.3/43.7			
	NC (28)	–	–			
PRJNA633959 (14)	AD dementia (100)	74.1 ± 9.2	57.0/43.0	V3–V4	Paired	Lishui, China
	NC (71)	73.1 ± 7.8	50.7/49.3			
PRJNA489760 (15)	AD dementia (30)	66.3 ± 5.1	50.0/50.0	V3–V4	Paired	Shanghai, China
	MCI (30)	65.4 ± 7.6	60.0/40.0			
	NC (30)	63.9 ± 5.1	56.7/43.3			
PRJNA611839 (16)	AD dementia (21)	76.2 (9.9)	38.1/61.9	V3–V4	Paired	Shanghai, China
	NC (44)	78.4 (6.6)	54.5/45.5			
PRJNA554111 (17)	AD dementia (43)	70.1 ± 8.8	46.5/53.5	V3–V4	Paired	Chongqing, China
	NC (43)	69.7 ± 9.2	46.5/53.5			
PRJNA946900 (18)	AD dementia (225)	–	–	V4–V5	Paired	Beijing, China
	MCI (38)	69.1 ± 7.2	60.5/39.5			
	NC (175)	–	–			
PRJNA792014 (19)	AD dementia (13)	72.0 ± 4.50	53.8/46.2	V3–V4	Paired	Shandong, China
	NC (13)	71.3 ± 6.30	61.5/38.5			
PRJNA855571 (20)	AD dementia (125)	–	–	V3–V4	Single	Fujian, China
	NC (40)	70.0 (14.8)	60.0/40.0			
PRJNA690972 (21)	MCI (13)	–	–	V3–V4	Single	Beijing, China
	NC (33)	–	–			
PRJNA811324 (22)	AD dementia (41)	68.0 (12.0)	73.2/26.8	–	Paired	Nur-Sultan, Kazakhstan
	NC (43)	68.0 (14.0)	81.4/18.6			
PRJNA734525 (23)	AD dementia (47)	71.4 ± 5.1	48.9/51.1	V3–V4	Paired	Istanbul, Turkey
	MCI (27)	69.2 ± 6.4	40.7/59.3			
	NC (51)	67.0 ± 5.3	45.1/54.9			
PRJEB51982 (12)	AD dementia (25)	71.3 ± 7.3	68.0/32.0	V4	Paired	Wisconsin, USA
	NC (25)	69.3 ± 7.5	72.0/28.0			
PRJNA801673 (24)	MCI (30)	64.7 ± 5.7	93.3/6.7	V4	Paired	Chicago, IL, USA
	NC (30)	64.9 ± 5.7	93.3/6.7			
PRJNA770746 (25)	AD dementia (45)	74.0 (13.0)	33.3/66.7	V4	Paired	Vancouver, Canada
	NC (54)	70.0 (8.0)	33.3/66.7			
PRJEB86129 (26)	AD dementia (51)	–	–	V4	Single	Puerto Rico, USA
	NC (51)	–	–			

^*a*^ Age iss presented as mean ± standard deviation or median (interquartile range).The symbol “–” indicates that no relevant information could be obtained from the study. AD: Alzheimer's disease; MCI: mild cognitive impairment; NC: normal control; F: female; M: male.

### Differences in gut microbiota composition

Microbiota composition analysis was used to identify bacterial genera with a relative abundance exceeding 1%. *Bacteroides* had the highest relative abundance in all groups, with the AD dementia group showing a lower abundance compared with the MCI and NC groups (AD dementia vs MCI vs NC: 10.0% vs 15.5% vs 13.9%, all *P* < 0.001), and the MCI group having the highest *Bacteroides* abundance (Fig. S2). *Faecalibacterium* was also less abundant in the AD dementia group than the MCI and NC groups, with the MCI group having the highest abundance (AD dementia vs MCI vs NC: 6.0% vs 10.3% vs 8.3%, all *P* < 0.001, Fig. S2). The AD dementia group had a higher *Escherichia-Shigella* (AD dementia vs MCI vs NC: 7.4% vs 5.5% vs 6.4%) and *Bifidobacterium* (AD dementia vs MCI vs NC: 6.4% vs 4.5% vs 4.6%) abundance than the MCI and NC groups (all *P* < 0.001, Fig. S2). *Subdoligranulum* was more abundant in the MCI group than in the NC group (MCI vs NC: 5.4% vs 4.9%, *P* = 0.007, Fig. S2), without statistical differences between the AD dementia and MCI groups (*P* = 0.120) or between the AD dementia and NC groups (*P* = 0.338).

### Differences in gut microbiota diversity

**Fig 2:**
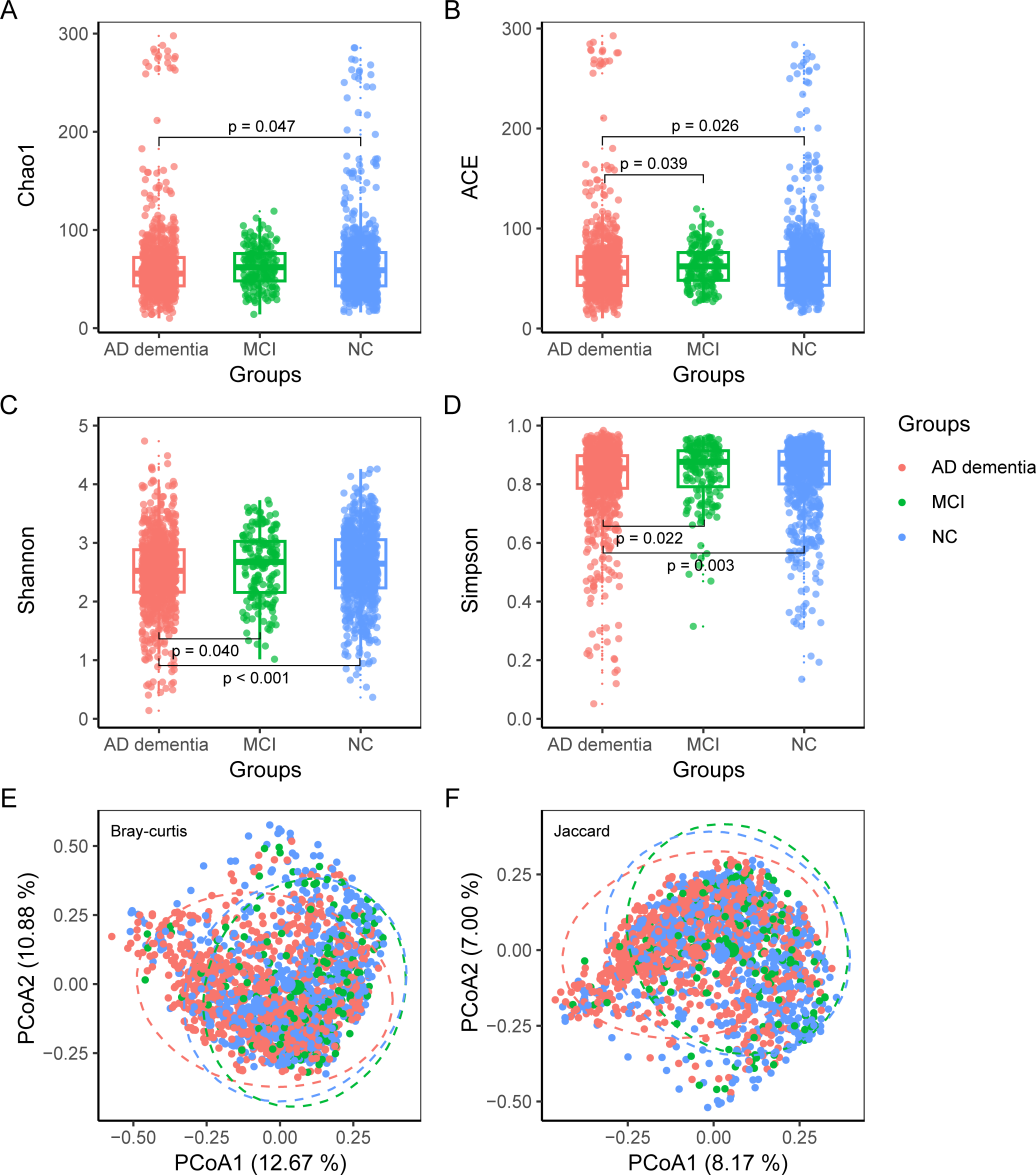
Comparison of diversity among the AD dementia, MCI, and NC groups. The indices of Chao1 (**A**), ACE (**B**), Shannon (**C**), and Simpson (**D**) were used to evaluate gut microbiota α-diversity in the three groups. Differences in α-diversity among the three groups were tested using the Kruskal-Wallis rank sum test, followed by a pairwise comparison using Dunn’s test. Bray-Curtis (**E**) and Jaccard (**F**) dissimilarity were used to evaluate the β-diversity of the three groups of gut microbiota. The Adonis test was used to test for differences in β-diversity among the three groups, and pairwise comparison showed statistically significant differences in β-diversity among all three groups. AD: Alzheimer’s disease; MCI: mild cognitive impairment; NC: normal control; Chao1: Chao species richness estimator; ACE: abundance-based coverage estimator.

Diversity analysis was conducted to calculate α-diversity indices and β-diversity indices. The AD dementia group had lower α-diversity than the NC group according to the Chao species richness estimator (Chao1) index (*P* = 0.047), and there was no statistical difference between the other two groups (Fig. 2). The AD dementia group had lower α-diversity than both the MCI and NC groups according to the abundance-based coverage estimator (ACE), Shannon, and Simpson indices (all *P* < 0.05), while there was no significant difference between the MCI and NC groups. Both Bray-Curtis and Jaccard dissimilarity indices indicated significant β-diversity differences among the three groups (*P* = 0.001). Specifically, the AD dementia group exhibited the greatest inter-individual microbial dissimilarity, whereas the MCI group showed the smallest (Fig. S3).

### Differences in the interactions of gut microbiota

**Fig 3:**
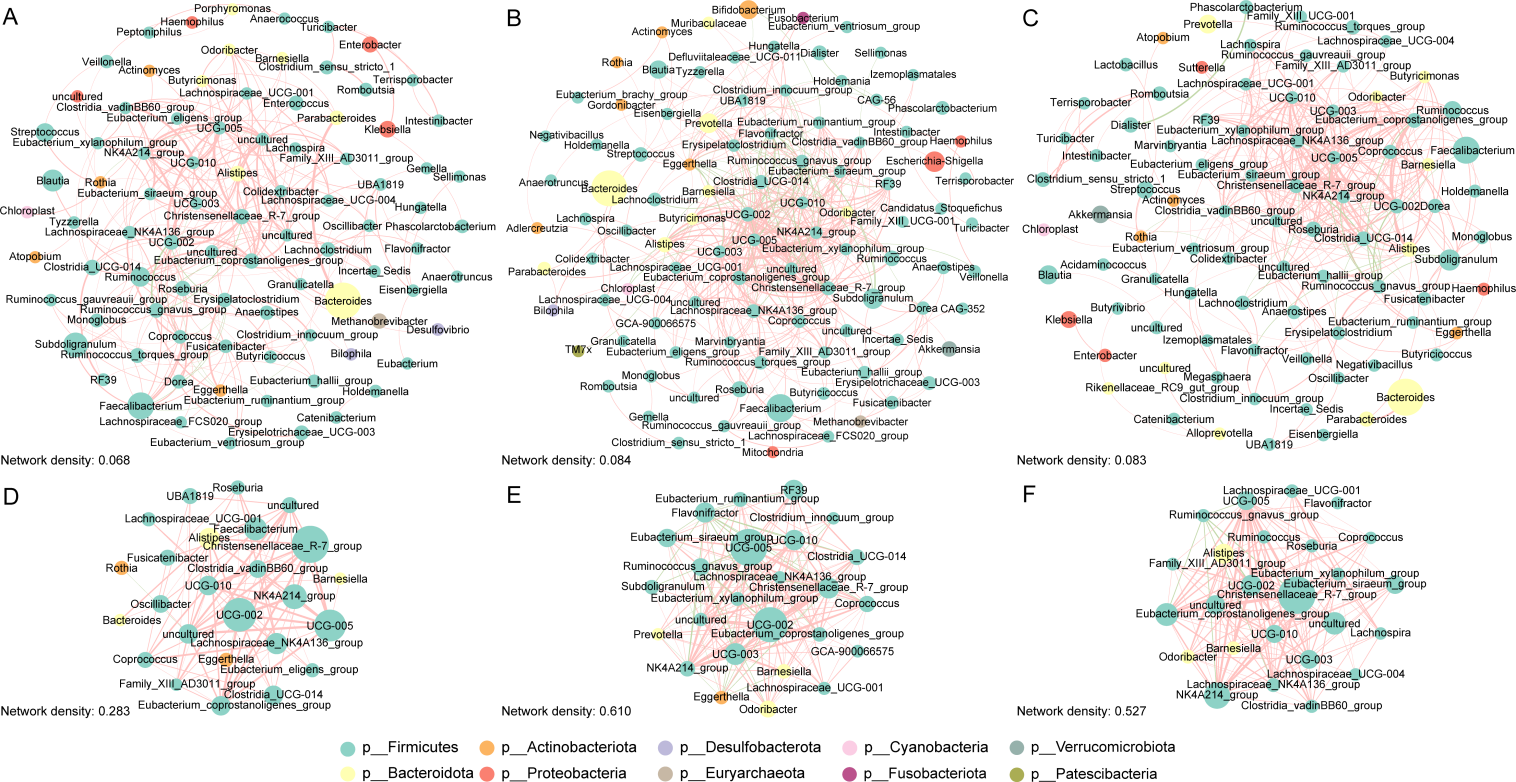
Correlation network of gut microbiota at the genus level in the AD dementia, MCI, and NC groups. Correlations between gut microbiota from the AD dementia group (**A**), MCI group (**B**), and NC group (**C**) were calculated using the sparse compositional correlation algorithm, and networks were constructed using correlation pairs with absolute values of correlation coefficients >0.3 and *P* < 0.05. Red lines indicated positive correlations, green lines indicated negative correlations, and circle size indicated relative abundance. The top 25 bacterial genera were selected as hub microbiota using the CytoHubba plugin with the maximal clique centrality (MCC) algorithm and subnetworks were constructed for the AD dementia group (**D**), MCI group (**E**), and NC group (**F**). The size of the circles represented the MCC values. AD: Alzheimer’s disease; MCI: mild cognitive impairment; NC: normal control.

The interactions among the gut microbiota in each group were analyzed through network analysis. The gut microbiota correlation networks in the AD dementia group (Fig. 3A), MCI group (Fig. 3B), and NC group (Fig. 3C) showed that roughly 78% of the bacterial genera belonged to the *Firmicutes*. Additionally, about 92% of the bacterial genera exhibited positive correlations, and 147 pairs of bacterial genera overlapped across the three groups. The correlation between certain pairs, such as *Clostridium innocuum group* and *Hungatella*, *Christensenellaceae R-7 group* and *Eubacterium coprostanoligenes group*, and *Roseburia* and *UCG-002*, gradually decreased across the NC, MCI, and AD dementia groups (Table S1). Compared with the NC and MCI groups, the network density of the AD dementia group decreased by 1.5% and 1.6%, respectively (network density: 0.083 vs 0.084 vs 0.068; Fig. 3A through C). Additional topology metrics were presented in Table S2. In the subnetwork (Fig. 3), 11 bacterial genera, including *UCG-010*, *UCG-002*, and *Christensenellaceae R-7 group*, co-occurred with high maximal clique centrality (MCC) values but low relative abundance values in the three groups (Fig. 3A through C).

### Identification of candidate gut microbiota indicators and construction of discriminative models

**Fig 4:**
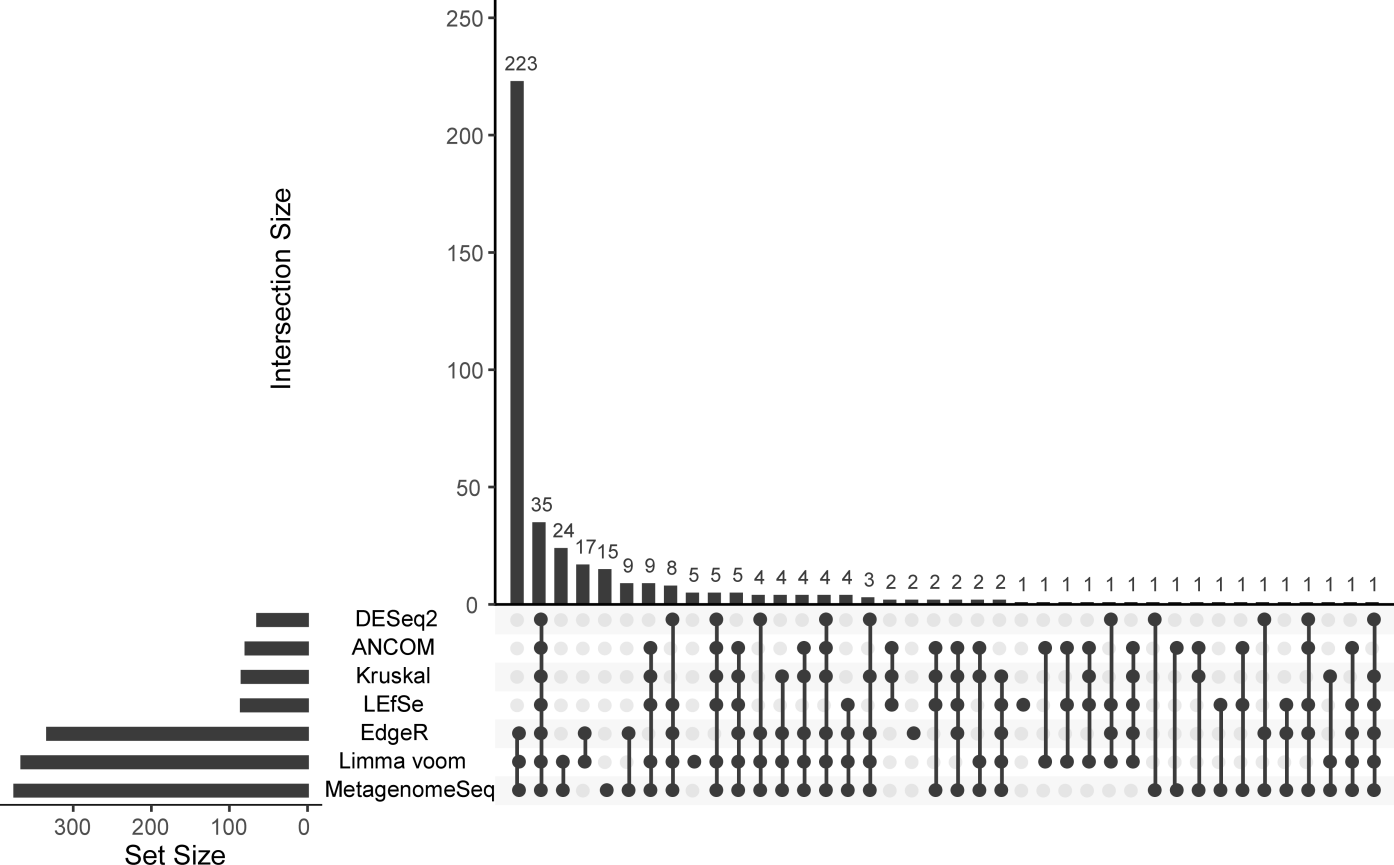
An UpSet plot of seven methods to identify candidate gut microbiota indicators. The black dots and connecting lines in the matrix below represented the intersection of the methods used in the discovery of candidate indicators, and the numbers on the bars indicated the results of the overlapping candidate indicators obtained by the corresponding methods. ANCOM: analysis of composition of microbiomes; Kruskal: Kruskal-Wallis rank sum test; LEfSe: linear discriminant analysis effect size analysis.

Differential bacterial genera identified simultaneously by seven common methods served as candidate indicators. The number of bacterial genera with intergroup differences reported by seven methods varied widely, with 35 bacterial genera found by all seven methods simultaneously (Fig. 4). These 35 bacterial genera were utilized as candidate gut microbiota indicators for identifying AD. Among them, *Methanobrevibacter*, *Olsenella*, *Collinsella*, *Erysipelatoclostridium*, *Enterococcus*, *Lactobacillus*, *Eubacterium*, *Eisenbergiella*, *Sellimonas*, *Peptoniphilus*, *Enterobacter*, *Acinetobacter*, *Akkermansia*, and *Oligella* exhibited higher relative abundance in the AD dementia group compared to the NC group (Fig. S4; Table S3). Conversely, the remaining candidate indicators showed lower relative abundance in the AD dementia group.

**Fig 5:**
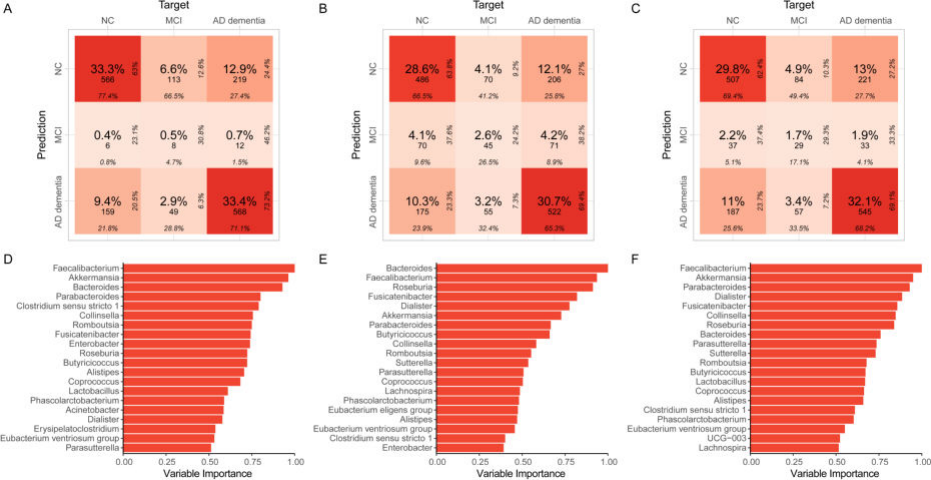
Results of three discriminative models used to identify AD in the overall population based on the 5-fold cross-validation method, random forest (RF), gradient boosting machine (GBM), and extreme gradient boosting (XGBoost) were used to build discriminative models for AD identification. (**A–C**) The confusion matrix plot of the predictive accuracy of the RF model, GBM model, and XGBoost model on the test sets, respectively. (**D–F**) The variable importance ranking (top 20) plots of the RF model, GBM model, and XGBoost model, respectively. AD: Alzheimer’s disease; MCI: mild cognitive impairment; NC: normal control.

Discriminative models for identifying AD were constructed using random forest (RF), gradient boosting machine (GBM), and extreme gradient boosting (XGBoost), based on the 35 identified candidate indicators. The RF model (Fig. 5A) predicted participants in the AD dementia and NC groups with test set accuracies of 71.1% and 77.4%, respectively, in the overall population. The GBM model (Fig. 5B) and XGBoost model (Fig. 5C) had slightly lower accuracies for the AD dementia group and NC group but performed better for the MCI group (26.5% and 17.1% respectively) compared with the RF model. Evaluation metrics showed that the RF model outperformed the GBM and XGBoost models whether the model was constructed in the overall population or in other populations (Table 2). The area under the receiver operating characteristic curve (AUC) value of the RF model in the overall population was 0.773, and the sensitivity and specificity were 0.705 and 0.728, respectively. The RF model constructed in the Chinese population outperformed the models constructed in the “Turkish and Kazakh” and “American and Canadian” populations (AUC: 0.826 vs 0.770 vs 0.628). Combining variable importance rankings across models in the overall population, *Faecalibacterium*, *Akkermansia*, *Bacteroides*, *Parabacteroides*, and *Fusicatenibacter* were the top five most important candidate indicators (Fig. 5D through F; Table S4).

**TABLE 2:** Performance of different discriminative models on the test set^*a*^

Groups	Model	Sensitivity	Specificity	PPV	NPV	Accuracy	F1 score	AUC (95%CI)
Overall population (*N* = 1,700)	RF	0.705	0.728	0.545	0.840	0.724	0.575	0.773 (0.754–0.794)
Overall population (*N* = 1,700)	GBM	0.655	0.729	0.519	0.801	0.699	0.543	0.734 (0.714–0.758)
Overall population (*N* = 1,700)	XGBoost	0.672	0.721	0.518	0.808	0.701	0.549	0.744 (0.725–0.765)
Chinese population (*N* = 1,180)	RF	0.820	0.726	0.574	0.875	0.726	0.614	0.826 (0.802–0.846)
Chinese population (*N* = 1,180)	GBM	0.722	0.759	0.566	0.839	0.732	0.588	0.772 (0.746–0.798)
Chinese population (*N* = 1,180)	XGBoost	0.799	0.731	0.562	0.864	0.735	0.611	0.803 (0.782–0.825)
Turkish and Kazakh population (*N* = 209)	RF	0.718	0.712	0.522	0.798	0.695	0.574	0.770 (0.710–0.817)
Turkish and Kazakh population (*N* = 209)	GBM	0.655	0.744	0.534	0.780	0.707	0.570	0.747 (0.685–0.800)
Turkish and Kazakh population (*N* = 209)	XGBoost	0.779	0.591	0.465	0.804	0.643	0.556	0.729 (0.676–0.786)
American and Canadian population (*N* = 311)	RF	0.726	0.519	0.417	0.779	0.611	0.506	0.628 (0.563–0.682)
American and Canadian population (*N* = 311)	GBM	0.698	0.481	0.394	0.776	0.581	0.483	0.575 (0.522–0.631)
American and Canadian population (*N* = 311)	XGBoost	0.599	0.562	0.393	0.731	0.577	0.442	0.568 (0.512–0.624)

^*a*^ Macro averages were used to calculate all metrics. PPV: positive predictive value; NPV: negative predictive value; AUC: area under the receiver operating characteristic curve; CI: confidence interval; RF: random forest; GBM: gradient boosting machine; XGBoost: extreme gradient boosting.

### Sensitivity analysis of candidate gut microbiota indicators

Sensitivity analysis was performed to explore the variation in the performance of models constructed using gut microbiota found simultaneously by five or more methods. After identifying candidate indicators of the gut microbiota by five or more methods, a total of 78 candidate indicators were identified by excluding the three bacterial genera for which genus names were not annotated (Fig. 4; Table S5). In contrast to the 35 shared candidate indicators identified by all seven methods, the models constructed from these candidate indicators showed similar performance on the test set. The AUC value of the RF model in the overall population was 0.791, and the sensitivity and specificity were 0.747 and 0.707, respectively. The RF model constructed in the Chinese population also outperformed the models constructed in the “Turkish and Kazakh” and “American and Canadian” populations (AUC: 0.850 vs 0.789 vs 0.607).

### Subgroup analysis of candidate gut microbiota indicators

Subgroup analysis was employed to investigate differences in the candidate indicators and discriminative models across different populations. Comparing the discriminative models constructed using the candidate indicators found in separate populations, we found that the whole trend was consistent with the models constructed using the candidate indicators found in the overall population (Fig. S5 to S7; Table S6). The RF model constructed in the Chinese population also outperformed the models constructed in the “Turkish and Kazakh” and “American and Canadian” populations (AUC: 0.830 vs 0.794 vs 0.724). The whole performance was slightly increased over the models constructed using the candidate indicators found in the overall population. The variable importance results from the models showed that the key candidate indicators varied across populations (Table S7 to S9). *Akkermansia* was most important in the Chinese population, while *Collinsella* and *Actinomyces* were more important in the “Turkish and Kazakh” and “American and Canadian” populations, respectively. Comparing candidate indicators in each subgroup with those in the overall population showed that candidate indicators in the Chinese population had the highest frequency, reaching 77% in the overall population (Table S10).

## DISCUSSION

In a large, multi-population study, we investigated the gut microbiota characteristics of AD patients and identified candidate indicators for AD diagnosis. Differences in gut microbiota composition were noted among the AD dementia, MCI, and NC groups, with the AD dementia group exhibiting lower α-diversity and greater inter-individual microbial dissimilarity. In addition, the AD dementia group displayed a simpler gut microbiota interaction network, suggesting a weakened robustness and resilience of the microbial communities. The 35 identified candidate gut microbiota indicators showed a significant ability to discriminate between the NC and AD dementia groups, suggesting their potential utility as non-invasive biomarkers for AD diagnosis.

We observed a lower α-diversity in the AD dementia group compared with the MCI and NC groups, consistent with previous findings (12, 13). This finding suggested a potential association between a reduced α-diversity and AD progression. Factors such as therapeutic drugs and dietary changes may also contribute to gut microbiota alterations. Greater inter-individual microbial dissimilarity was exhibited in the AD dementia group than in the other two groups, indicating a more heterogeneous gut microbiota composition among participants in the AD dementia group. While our observation of greater microbial dissimilarity in AD dementia aligns with the findings of Guo et al. (27), it contrasts with several other studies that reported no significant difference compared with NC groups (25, 28). These discrepancies might be attributed to key differences in study design, such as our use of a multi-population data set, larger sample size, and distinct sequencing methodologies. Our findings suggest that increased inter-individual microbial dissimilarity could be a relevant feature of AD. Nevertheless, future multi-center studies with standardized designs are warranted to validate this association.

The gut microbiota interaction network with strong correlation coefficients was sparser in the AD dementia group than in the NC group. Taken together with the greater inter-individual dissimilarity, these findings suggest a microbial community characterized by a loss of stable inter-microbial relationships. Similar findings were reported by Ling et al. (14), where the healthy control group exhibited a more complex gut microbiota interaction network. Notably, bacterial genera at the core of the network, such as *UCG-010*, *UCG-002*, and *Christensenellaceae R-7 group*, had relatively low abundance, suggesting their significant role in maintaining human health despite their low prevalence in the gut. The diminishing correlation between certain bacterial genera, such as *Clostridium innocuum group* and *Hungatella*, in the MCI and AD dementia groups compared to the NC group implies a potential association with the severity of AD.

We identified 35 bacterial genera as candidate indicators. A large number of these candidate indicators, including *Faecalibacterium*, *Bacteroides*, *Parabacteroides*, *Fusicatenibacter*, and *Roseburia*, have been reported in previous studies and exhibit lower relative abundance in the AD dementia patients than healthy individuals (14, 29, 30). In contrast, *Akkermansia*, *Collinsella*, *Lactobacillus*, *Enterobacter*, and *Enterococcus* demonstrated higher relative abundance in the AD dementia group than in the NC group in prior research (14, 19, 31). These findings suggest that these bacterial genera could serve as candidate indicators for diagnosing AD and tracking its progression, presenting opportunities for early intervention. In addition, our study identified *RF39* and *Oligella* to be two candidate indicators for AD, and they have not been reported in previous observational population studies.

The genus *RF39* is classified under the order *RF39* within the class *Bacilli* in the phylum *Firmicutes* (32). Although research on *RF39* is limited, one study suggests that it is a major lineage in the human gut microbiota, with the potential to produce acetate and hydrogen (33). Acetate is a short-chain fatty acid with neuroprotective properties (34). It can cross the blood-brain barrier and is taken up by the brain, providing an important source of energy for glial cells (35). In a mouse model of AD, acetate was discovered to exert anti-neuroinflammatory effects by upregulating G-protein-coupled receptor 41 and inhibiting the ERK/JNK/NF-κB pathway (36). In our study, the AD dementia group showed a decreased relative abundance of *RF39*. This reduction may result in lower levels of acetate and hydrogen, diminishing their protective effects on cognitive function and potentially influencing the development of AD.

*Oligella* is a gram-negative pathogenic bacterium known to cause infections such as pulmonary abscess, urosepsis, and knee septic arthritis in humans (37). Our study found a higher relative abundance of *Oligella* in the AD dementia group compared to the NC group. Similarly, a preterm rat model with cognitive impairment showed higher levels of *Oligella* than the normal cognitive group (38). No studies have explored the relationship between *Oligella* and AD, although it may be linked to the inflammatory response triggered by *Oligella*. Intestinal inflammation is associated with brain pathology and could worsen AD progression (39). However, the specific mechanism by which *Oligella* influences AD requires further investigation.

We found that RF models constructed using candidate indicators identified from the overall, Chinese, “Turkish and Kazakh,” and “American and Canadian” populations each achieved AUC values above 0.7 in their respective cohorts, demonstrating their potential for clinical application. The variable importance analysis revealed that the top-ranking genera differed across populations. *Faecalibacterium* was the most important genus in the overall population, *Akkermansia* ranked highest in the Chinese population, *Collinsella* in the “Turkish and Kazakh” population, and *Actinomyces* in the “American and Canadian” population. These results offer a valuable reference for selecting specific gut microbiota for different application scenarios.

*Faecalibacterium* is a genus of butyrate-producing bacteria known for its anti-inflammatory properties, achieved through butyrate production and the induction of a tolerogenic cytokine profile (14). Sheng et al. reported that a reduction in *Faecalibacterium* abundance was associated with increased Aβ deposition in the brain, potentially contributing to the progression of AD (40). *Akkermansia* is a genus of propionate-producing bacteria that supports gut health and overall metabolic function (41). However, Ling et al. found that an increased abundance of *Akkermansia* was associated with lower cognitive function scores and reduced levels of anti-inflammatory cytokines, suggesting a potentially detrimental role in the development of AD in older adults (14). *Collinsella* has been shown to impair intestinal barrier function by reducing the expression of tight junction proteins in intestinal epithelial cells, leading to the translocation of lipopolysaccharides into the circulatory system and triggering an inflammatory response (42). Additionally, one study reported a positive correlation between *Collinsella* abundance and the APOE rs429358 risk allele, suggesting that their interaction may accelerate the progression of AD (43). *Actinomyces* is a genus associated with chronic inflammation, which may contribute to the development of AD through inflammatory pathways (44). Zhu et al. also found that *Actinomyces* was more abundant in patients with AD compared to cognitively normal individuals (28).

Several candidate microbial indicators identified in our study overlap with those reported in other neurodegenerative disorders. For instance, genera such as *Faecalibacterium*, *Fusicatenibacter*, and *Roseburia* have also been found diminished in individuals with Parkinson’s disease (45). Similarly, *Akkermansia* and *Collinsella* have been observed to be enriched in both Parkinson’s disease and dementia with Lewy bodies (46, 47). Given that we focused exclusively on the AD without including other neurodegenerative disorders, we cannot definitively establish the specificity of these indicators to AD. Therefore, disentangling disease-specific signatures from those reflecting general neurological decline is essential in the future. Our findings provide a foundational rationale for subsequent research that compares the gut microbiota across multiple etiologies of cognitive impairment, representing a critical step toward validating robust, clinically specific diagnostic biomarkers.

Our study also carries important implications for public health. The identified candidate indicators represent a promising foundation for future research into the non-invasive, early diagnosis of AD across diverse populations. Although external validation is essential, these results could accelerate the development of novel, adaptable protocols suitable for community-wide screening.

This study has several limitations. First, all the included studies utilized 16S rRNA gene sequencing, limiting the accurate annotation of gut microbiota to the genus level rather than the species level. Second, the absence of data on key confounders, such as race, genetic factors, diet, and medication, in the public data set prevented the adjustment for these variables, potentially introducing bias into the results. Third, despite our intention to explore the relationship between AD and gut microbiota across multiple populations, the predominance of Chinese samples may have biased the findings toward characteristics specific to the Chinese population. Fourth, the smaller sample size in the MCI group compared with the other groups may have introduced bias, despite the use of class balancing during model development. Fifth, all the included studies were cross-sectional, preventing the establishment of a causal relationship between AD and gut microbiota.

In summary, our study demonstrates that the AD dementia group consistently showed lower α-diversity and a sparser microbiota interaction network than the other groups. Discriminative models based on 35 identified candidate microbiota indicators showed promising accuracy for AD identification. These findings contribute to the development of non-invasive biomarkers for AD diagnosis and targeted microbiota therapies and provide a valuable reference for selecting specific biomarkers for different application scenarios.

## MATERIALS AND METHODS

### Data collection

We collected data through two approaches (Fig. 1). First, a systematic search of the published literature was performed in the PubMed database for relevant studies on AD and gut microbiota. The public data reported in these studies were used as the data source for our study. Second, a direct search was conducted in the National Center for Biotechnology Information (NCBI) database to access the public data related to AD and gut microbiota stored within the system. In this study, AD primarily referred to MCI and dementia stages caused by AD.

During the literature collection process, we searched from the inception of PubMed to 22 April 2025, the search formula and inclusion/exclusion criteria were outlined in File S1. In the preliminary selection based on titles and abstracts, 56 items were selected from the initial pool of 1,893 items. After reviewing the full text, 12 items were identified for inclusion. During data collection in the NCBI database, we searched using AD-related keywords (“Alzheimer,” “AD,” “Cognitive Impairment,” and “MCI”) and microbiota-related keywords (“Microbiome,” “Microbiota,” and “Metagenome”) for the retrieval process. We searched from the inception of NCBI until 22 April 2025, primarily exploring data from the Bioproject, Biosample, and Sequence Read Archive (SRA) sections. We applied similar inclusion and exclusion criteria for data selection as the first method. After comparing and excluding duplicates from the two search methods, 15 items were included in the final data set for this study.

### Data processing

Fastq format files of raw sequencing data of the gut microbiota were downloaded from SRA using the SRA Toolkit tool (version 3.0.0) (48). Then, sequencing data were subjected to quality assessment using FastQC (version 0.11.9) (49). Next, FIGARO was utilized to determine the optimal parameters for quality control in the DADA2 analysis (50). Subsequently, the sequencing data underwent quality control using the DADA2 plugin (51) in the QIIME2 (52). The taxonomic annotation of the representative sequences was then performed using the Vsearch method (53, 54) in QIIME2, using Silva 138 (99% OTUs data set) as a reference database (55). At this point, the taxonomy table was obtained for the phylum to genus level. After completing the above process for the 15 studies, the genus level taxonomy table from each study was selected and combined. We initially combined the 15 tables by matching column names. Identical columns were combined directly, while those with different names were kept as separate columns, with missing data filled in with zeros. To minimize potential errors introduced by sequencing factors, we first excluded genera present in only a single sample, followed by the removal of genera with read counts <0.001% of the total sequenced reads. The combined data were subsequently processed using the *MMUPHin* package (version 1.20.0) in R to remove batch effects. This package is particularly well-suited for combining microbial data and can effectively remove batch effects across different studies (56). Principal coordinate analysis (PCoA) (57) and the Adonis test (58) were employed to assess the effectiveness of batch correction. The combined data consisted of three groups: AD dementia, MCI, and NC. All subsequent analyses relied on this combined data and predominantly used R.

### Gut microbiota characteristics analysis

Microbiota composition analysis identified bacterial genera with a relative abundance exceeding 1%, which were then visualized using bar charts created with the ggplot2 package (version 3.4.2). Diversity analysis was conducted using the vegan package (version 2.6-4) to calculate α-diversity indices, including Chao1, ACE, Shannon, and Simpson indices, as well as β-diversity indices (Bray-Curtis and Jaccard dissimilarity). Box plots were generated using the ggplot2 package to present the α-diversity indices. PCoA was used to present the β-diversity indices. The interactions among the gut microbiota in each group were analyzed using FastSpar (version 1.0.0) and the sparse compositional correlation algorithm (59), with *P*-values adjusted using the Benjamini-Hochberg correction. Gephi (version 0.9.2) (60) was used to visualize the network for pairs with absolute values of correlation coefficients >0.3 and *P* < 0.05, and to calculate the network density, which was defined as (2 × edges)/(nodes × (nodes − 1)) (61). CytoHubba plugin (version 0.1) in Cytoscape (version 3.9.1) (62) was used to calculate topological metrics, including MCC, density of maximum neighborhood component, degree, edge percolated component, closeness, radiality, betweenness, stress, and clustering coefficient. The MCC was subsequently employed to identify the top 25 bacterial genera as hub microbiota.

### Candidate gut microbiota indicator identification analysis

The microbiomeMarker package (version 1.4.0) (63) was used for candidate indicator analysis, employing seven common methods for candidate indicator identification provided in the package: Kruskal-Wallis rank sum test, DESeq2 (64), EdgeR (65), Voom (66), linear discriminant analysis effect size analysis (67), MetagenomeSeq (68), and analysis of composition of microbiomes (69). Differential bacterial genera identified simultaneously by seven methods were considered candidate gut microbiota indicators and presented using UpSet plots.

### Statistical analysis

The chi-square test was used to assess the differences in proportions of bacterial genera among the three groups after microbiota composition analysis, followed by a pairwise proportion test. The Kruskal-Wallis rank sum test was employed to evaluate differences in α-diversity indices among the groups, followed by Dunn’s test for pairwise comparisons. The Adonis test was used to examine differences in β-diversity indices among the groups, and pairwise comparisons were conducted using the pairwiseAdonis package (version 0.4.1). Violin plots were utilized to display the relative abundance of candidate gut microbiota indicators in different groups, and group differences were assessed using the Kruskal-Wallis rank sum test followed by Dunn’s test. All *P*-values for pairwise comparisons were adjusted using the Benjamini-Hochberg correction.

A 5-fold cross-validation approach was used to construct discriminative models for identifying AD using RF, GBM, and XGBoost methods. All models were trained with class weight adjustments to address class imbalance. Candidate indicators were used as independent variables, with the outcome variable serving as the dependent variable in each model. Confusion matrices were used to show the predictive effect, and bar charts were used to display the results of variable importance for each model. The performance of the models was evaluated using sensitivity, specificity, positive predictive value, negative predictive value, accuracy, F1 score, and AUC. Sensitivity analysis was performed to explore the variation in the performance of models constructed using gut microbiota found simultaneously by five or more methods described above. Subgroup analysis was employed to investigate differences in the candidate indicators and discriminative models across different populations. For all analyses, *P*-values < 0.05 or corrected *P*-values < 0.05 were considered statistically significant.

### STORMS checklist

The STORMS checklist for this study has been completed and is publicly available. It can be accessed via Figshare at https://doi.org/10.6084/m9.figshare.30859631

## Data Availability

This study used public data, with raw sequencing data accessible on the US National Center for Biotechnology Information website (https://www.ncbi.nlm.nih.gov/). Project numbers include PRJNA496408, PRJNA633959, PRJNA489760, PRJNA611839, PRJNA554111, PRJNA946900, PRJNA792014, PRJNA855571, PRJNA690972, PRJNA811324, PRJNA734525, PRJEB51982, PRJNA801673, PRJNA770746, and PRJEB86129. Additional data are provided within the article and supplemental material. The custom code and analysis scripts generated during this study are available from the corresponding author (or the first author) upon reasonable request.

## SUPPLEMENTAL MATERIAL

Supplemental material msystems.01754-25-s0001.pdf Supplemental methods, figures, and table.

ASM does not own the copyrights to Supplemental Material that may be linked to, or accessed through, an article. The authors have granted ASM a non-exclusive, world-wide license to publish the Supplemental Material files. Please contact the corresponding author directly for reuse.
