# Supplementary material for: Identifying candidate gut microbiota indicators for Alzheimer’s disease through integrated data
Source: mSystems. 2026 Mar 25;11(4):e01754-25. doi: 10.1128/msystems.01754-25 (PMC13098251; doi:10.1128/msystems.01754-25)
Supplement: Supplemental material — Supplemental methods, figures, and table. [file msystems.01754-25-s0001.pdf]

Identifying candidate gut microbiota indicators for Alzheimer's disease through integrated data

Jing Wang[1,2], Hanting Liu[3], Hao Lai[2], Yining Bao[2], Mingwang Shen[2,4], Chao Li[5], Lu Ma[6,4], Ting Wu[7], Siyu Yang[8], Xinyu Du[7], Terence J. O'Brien[9], Jing Zhang[7], Lei Zhang[1,2,9,10*]

1. Phase I clinical trial research ward, The Second Affiliated Hospital of Xi'an Jiaotong University, No.157 Xi Wu Road, Xi'an, Shaanxi 710004, China.
2. China-Australia Joint Research Centre for Infectious Diseases, School of Public Health, Xi'an Jiaotong University Health Science Centre, No. 76 Yanta West Road, Xi'an, Shaanxi 710061, China.
3. Department of Medical Statistics, School of Public Health, Sun Yat-sen University, No. 74 Zhongshan Second Road, Guangzhou, Guangdong 510080, China.
4. Key Laboratory for Disease Prevention and Control and Health Promotion of Shaanxi Province, No. 76 Yanta West Road, Xi'an, Shaanxi 710061, China.
5. Department of Epidemiology and Health Statistics, School of Public Health, Xi'an Jiaotong University Health Science Centre, No. 76 Yanta West Road, Xi'an, Shaanxi 710061, China.
6. School of Public Health, Xi'an Jiaotong University Health Science Centre, No. 76 Yanta West Road, Xi'an, Shaanxi 710061, China.
7. Department of Neurology, The First Affiliated Hospital of Nanjing Medical University, No.300 Guangzhou Road, Nanjing 210029, China.
8. Department of Neurology, The Second Hospital of Nanjing, No. 1-1 Zhongfu Road, Nanjing 210003, China.
9. The Department of Neuroscience, The School of Translational Medicine, Faculty of Medicine, Nursing and Health Sciences, Monash University & Alfred Health, 27 Rainforest Walk, Clayton, Victoria 3168, Australia.
10. Artificial Intelligence and Modelling in Epidemiology Program, Melbourne Sexual Health Centre, Alfred Health, 580 Swanston Street, Carlton, Victoria 3053, Australia.

*** Correspondence:**
Lei Zhang, Phase I clinical trial research ward, The Second Affiliated Hospital of Xi'an Jiaotong University, No.157 Xi Wu Road, Xi'an, Shaanxi 710004, China. Tel.: +86 15910593477; E-mail: Lei.zhang1@monash.edu.

Table of contents of the supplementary material

Supplementary file 1 ……………………………………………………………………………………3
Figure S1 ………………………………………………………………………………………….....4
Figure S2 ………………………………………………………………………………………….....5
Figure S3 ………………………………………………………………………………………….....6
Figure S4 ………………………………………………………………………………………….....7
Figure S5 ………………………………………………………………………………………….....8
Figure S6 ………………………………………………………………………………………….....9
Figure S7 …………………………………………………………………………………………10
Table S1 …………………………………………………………………………………………..11
Table S2 …………………………………………………………………………………………..16
Table S3 …………………………………………………………………………………………..25
Table S4 …………………………………………………………………………………………..29
Table S5 ……………………………………………………………………………………….…31
Table S6 ……………………………………………………………………………………….…32
Table S7 ……………………………………………………………………………………….…33
Table S8 ……………………………………………………………………………………….…35
Table S9 ………………………………………………………………………..……………36
Table S10 ……………………………………………………………………………………………38

Supplementary file 1: methods

Search Formulas in Pubmed

The search formulas used were as follows: (Alzheimer disease OR Senile Dementia OR Alzheimer Type Dementia OR Alzheimer Type Senile Dementia OR Primary Senile Degenerative Dementia OR Alzheimer Sclerosis OR Alzheimer Syndrome OR Alzheimer Dementia OR Acute Confusional Senile Dementia OR Presenile Dementia OR Late Onset Alzheimer Disease OR Focal Onset Alzheimer's Disease OR Familial Alzheimer Disease OR Early Onset Alzheimer Disease OR Presenile Alzheimer Dementia) AND (Gastrointestinal microbiome OR Gut Microbiome OR Gut Microflora OR Gut Microbiota OR Gastrointestinal Flora OR Gut Flora OR Gastrointestinal Microbiota OR Gastrointestinal Microbial Community OR Gastrointestinal Microflora OR Gastric Microbiome OR Intestinal Microbiome OR Intestinal Microbiota OR Intestinal Microflora OR Intestinal Flora OR Enteric Bacteria).

Inclusion and exclusion criteria

We excluded studies that: (1) involved intervention populations, (2) addressed other phenotypes, (3) did not focus on AD-related gut microbiota changes, (4) were conducted on animals, (5) were reviews or meta-analyses, (6) comprised additional types such as Mendelian randomisation and bibliometrics, (7) lacked data on gut microbiota sequencing, (8) used metagenomic shotgun sequencing, (9) employed internal transcribed spacer 2 rRNA gene sequencing, (10) lacked phenotype grouping data, or (11) studied about preclinical AD. Conversely, we retained studies that: (1) were observational population studies, or (2) utilised 16S rRNA gene sequencing.

Supplementary Figures

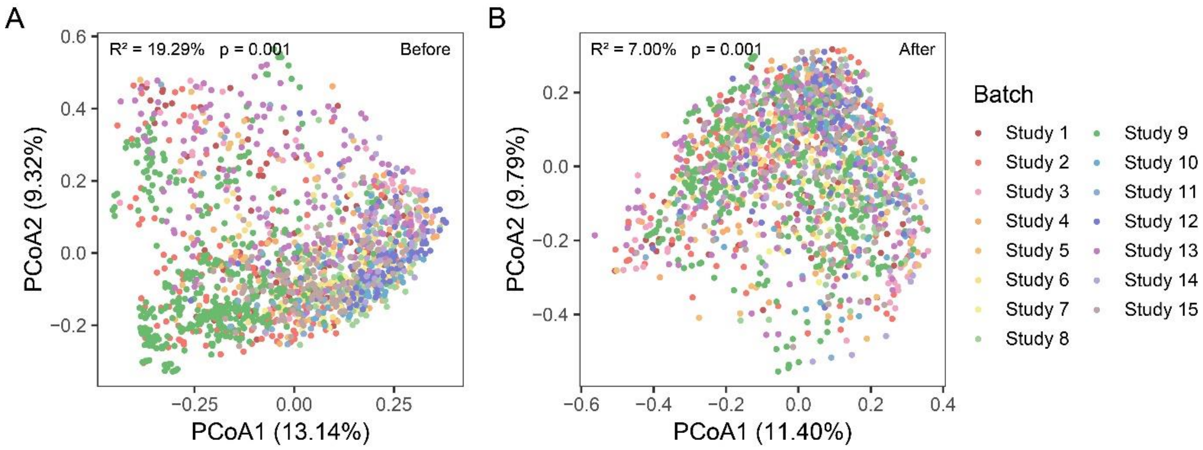

Supplementary Figure 1. Principal Coordinate Analysis (PCoA) of Bray–Curtis dissimilarity before and after batch effect correction. (A) Results prior to batch correction. (B) Results following batch correction. Statistical significance of batch effects was evaluated using the Adonis test.

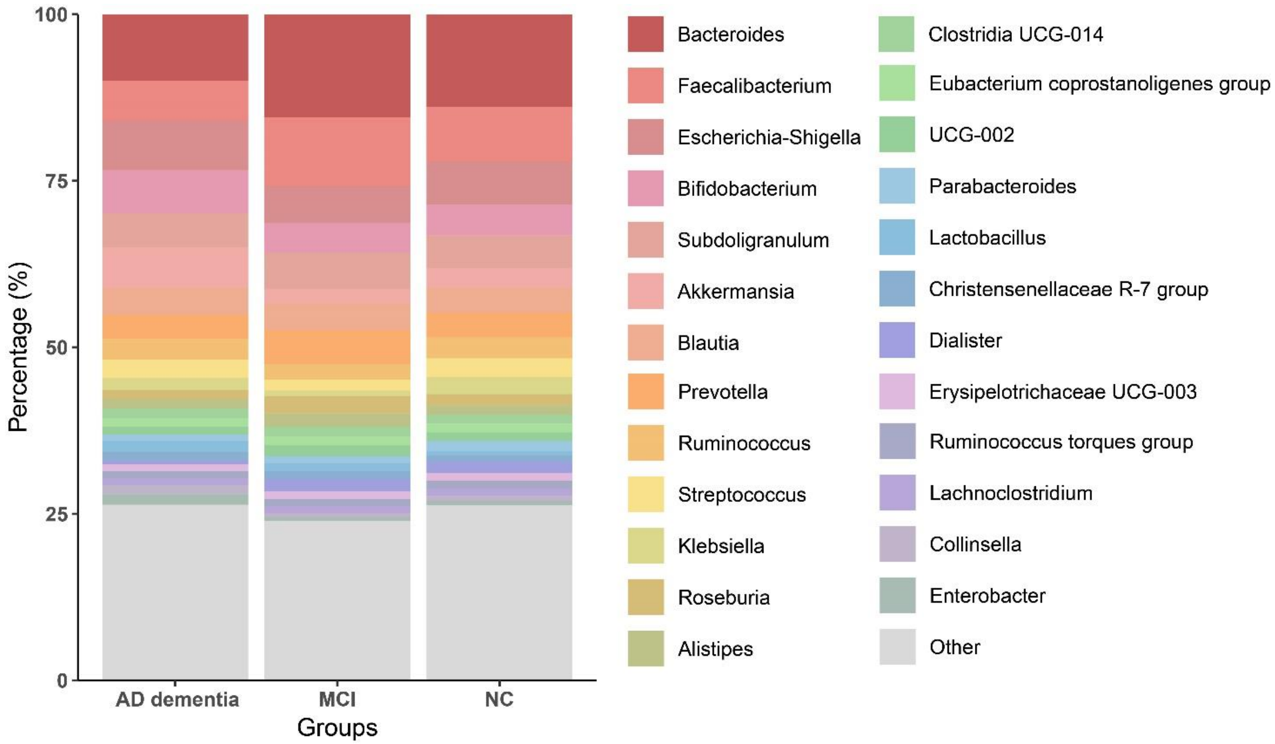

Supplementary Figure 2. Composition of gut microbiota at the genus level in the AD dementia, MCI, and NC groups. Bacterial genera with a relative abundance exceeding 1% were presented individually, whereas those with a relative abundance below 1% were combined and labelled as "Other". AD: Alzheimer's disease; MCI: Mild cognitive impairment; NC: Normal control.

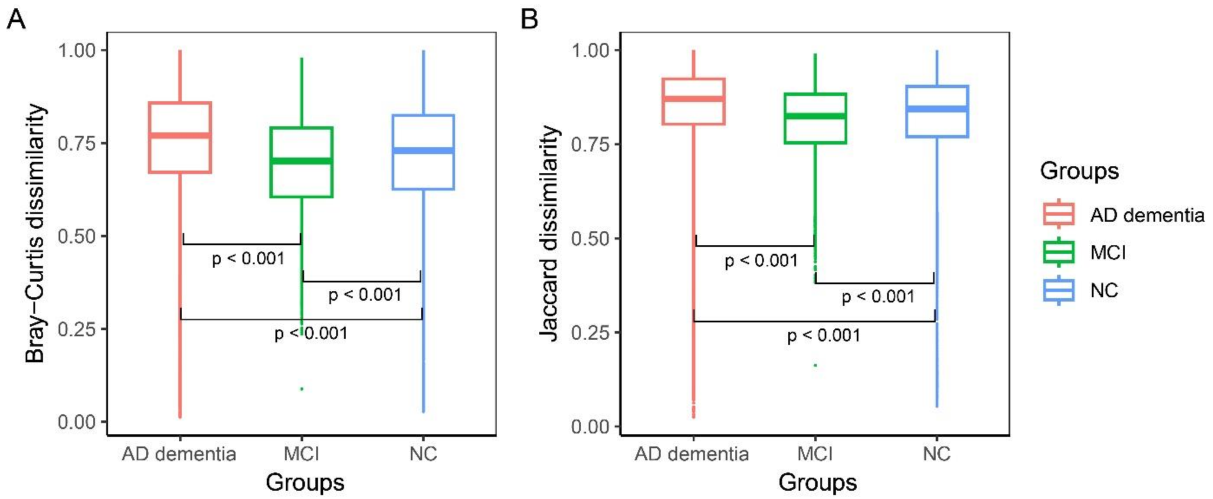

Supplementary Figure 3. Comparison of β-diversity among the AD dementia, MCI and NC groups. The differences in Bray-Curtis dissimilarity (A) and Jaccard dissimilarity (B) among the three groups were illustrated. Differences among the three groups were tested using the Kruskal-Wallis rank sum test, followed by a pairwise comparison using Dunn's test. AD: Alzheimer's disease; MCI: Mild cognitive impairment; NC: Normal control.

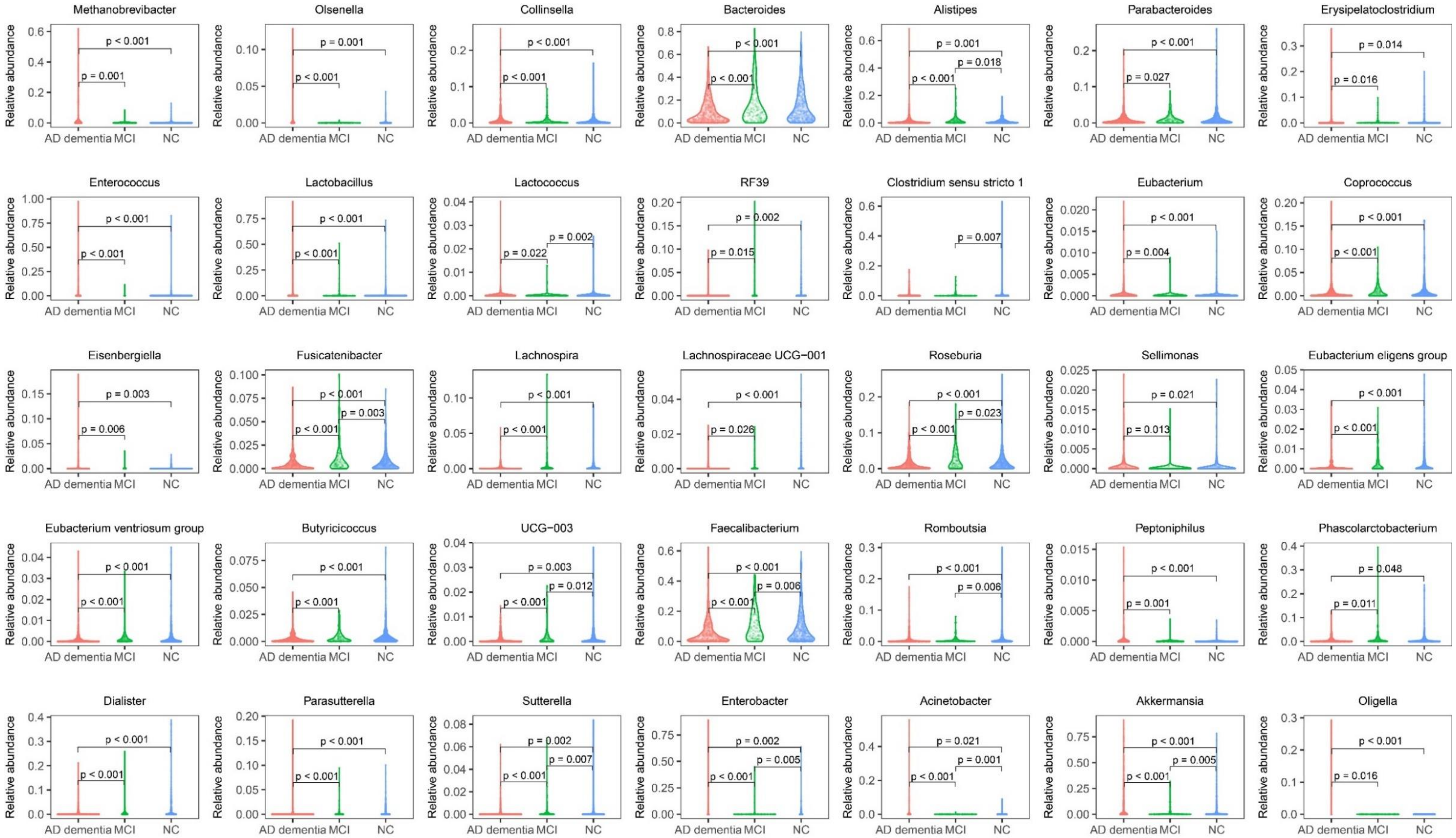

Supplementary Figure 4. Comparison of the relative abundance of candidate gut microbiota indicators among the AD dementia, MCI and NC groups. Differences among the three groups were tested using the Kruskal-Wallis rank sum test, followed by a pairwise comparison using Dunn's test. AD: Alzheimer's disease; MCI: Mild cognitive impairment; NC: Normal control.

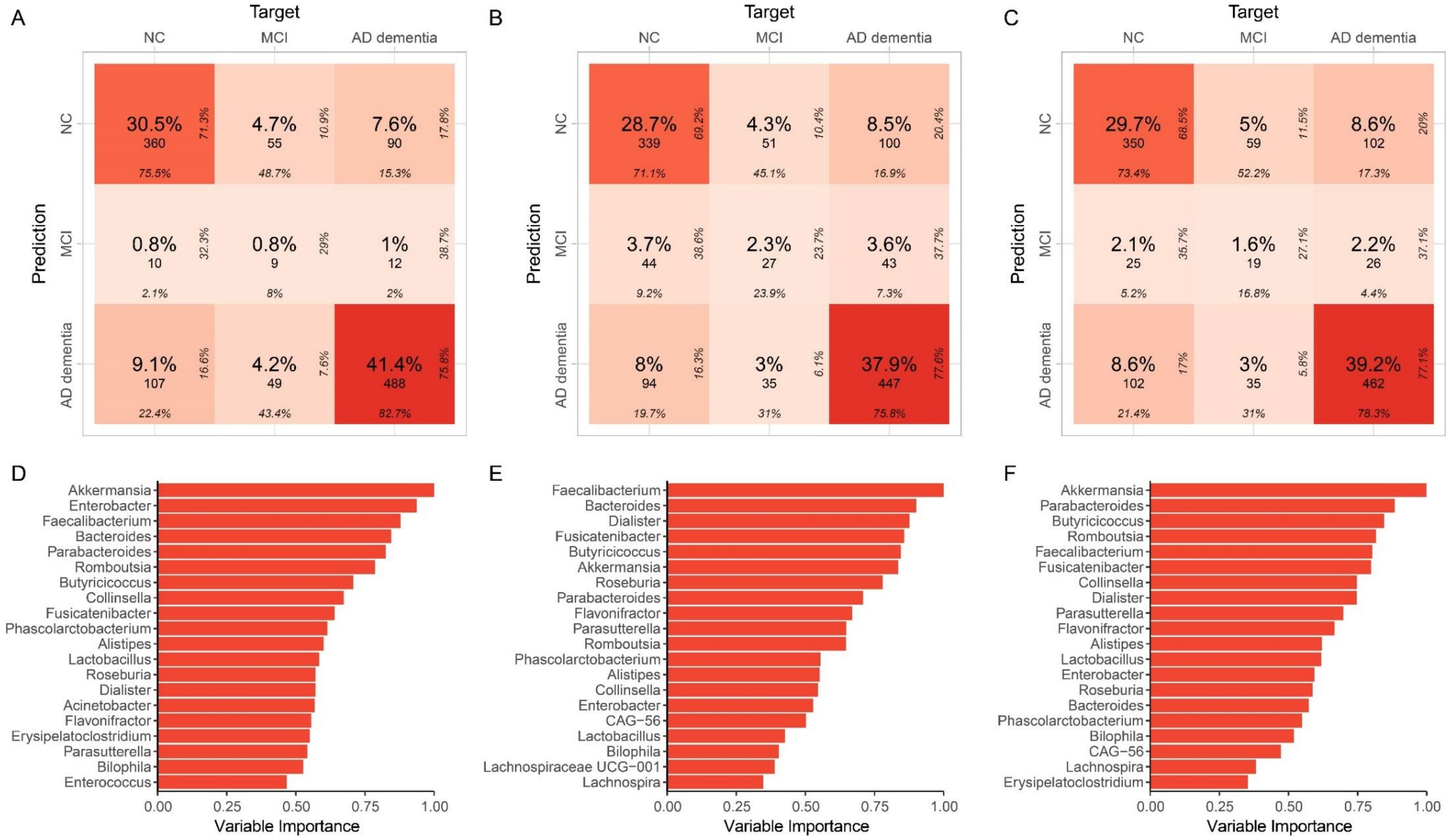

Supplementary Figure 5. Results of three discriminative models used to identify AD in the Chinese population. A-C were the confusion matrix plot of the predictive accuracy of the Random Forest (RF) model, Gradient Boosting Machine (GBM) model and Extreme Gradient Boosting (XGBoost) model on the test sets, respectively. D-F were the variable importance ranking (top twenty) plots of the RF model, GBM model and XGBoost model respectively. AD: Alzheimer's disease; MCI: Mild cognitive impairment; NC: Normal control.

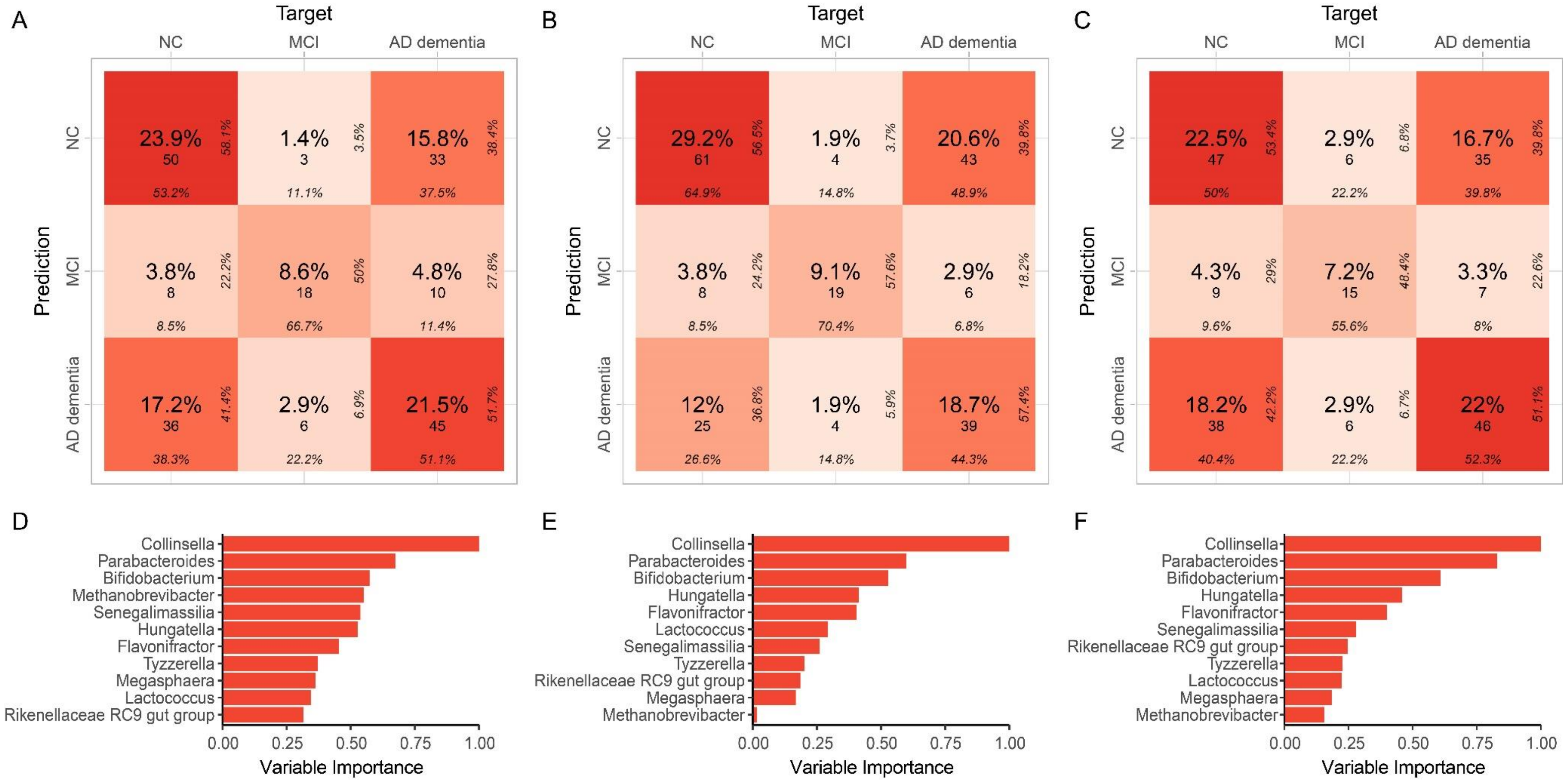

Supplementary Figure 6. Results of three discriminative models used to identify AD in the Turkish and Kazakh population. A-C were the confusion matrix plot of the predictive accuracy of the Random Forest (RF) model, Gradient Boosting Machine (GBM) model and Extreme Gradient Boosting (XGBoost) model on the test sets, respectively. D-F were the variable importance ranking plots of the RF model, GBM model and XGBoost model respectively. AD: Alzheimer's disease; MCI: Mild cognitive impairment; NC: Normal control.

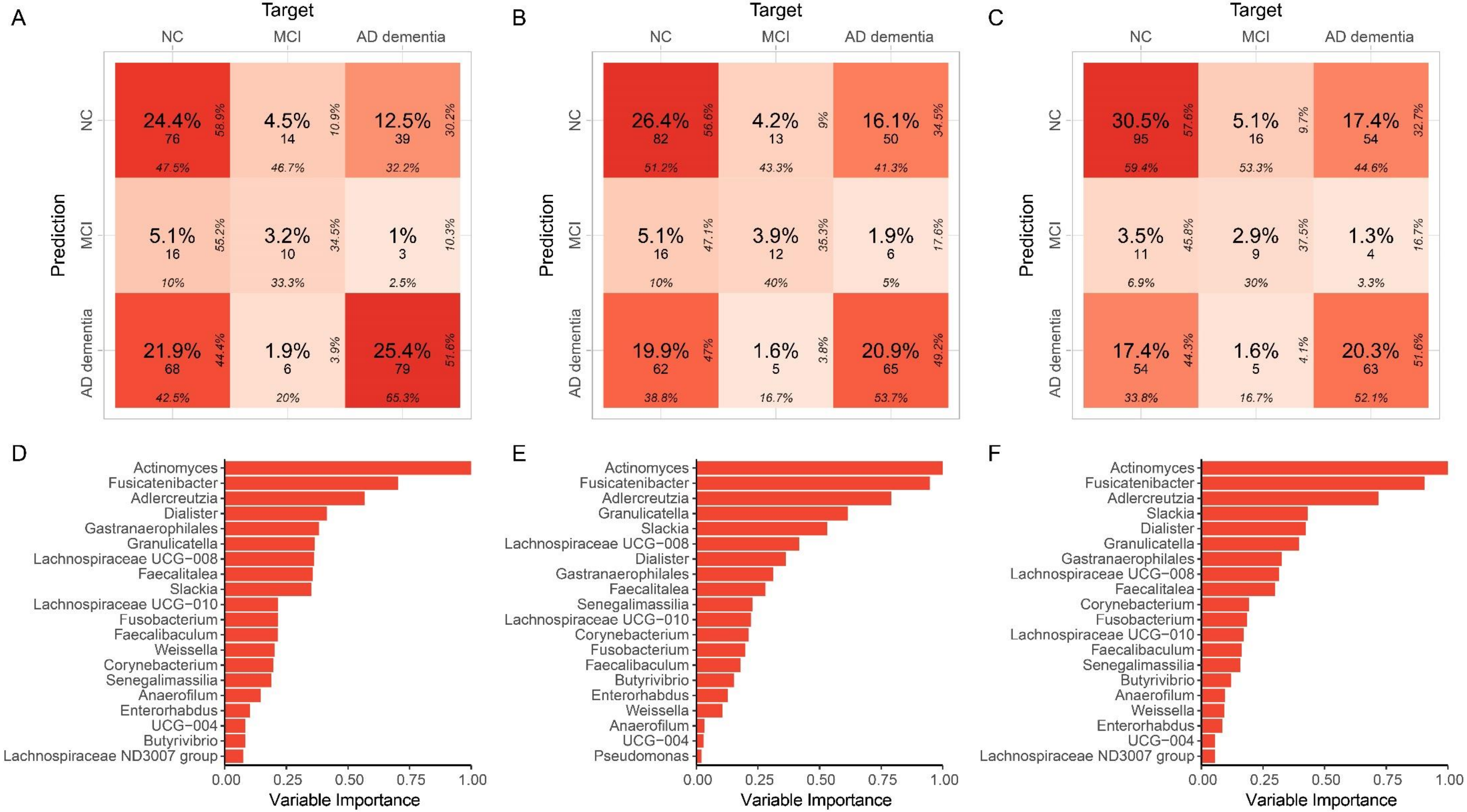

Supplementary Figure 7. Results of three discriminative models used to identify AD in the American and Canadian population. A-C were the confusion matrix plot of the predictive accuracy of the Random Forest (RF) model, Gradient Boosting Machine (GBM) model and Extreme Gradient Boosting (XGBoost) model on the test sets, respectively. D-F were the variable importance ranking (top twenty) plots of the RF model, GBM model and XGBoost model respectively. AD: Alzheimer's disease; MCI: Mild cognitive impairment; NC: Normal control.

Supplementary tables

Supplementary Table 1. Correlation coefficients for pairs co-occurring in the gut microbiota networks of the AD dementia, MCI, and NC groups

Source	Target	Correlation coefficients		
		AD dementia	MCI	NC
Rothia	Streptococcus	0.388	0.314	0.381
Eggerthella	Erysipelatoclostridium	0.490	0.461	0.368
Eggerthella	Ruminococcus gnavus group	0.426	0.375	0.409
Bacteroides	Parabacteroides	0.585	0.353	0.542
Barnesiella	Odoribacter	0.354	0.342	0.388
Barnesiella	Alistipes	0.322	0.360	0.338
Barnesiella	UCG-005	0.315	0.492	0.428
Barnesiella	UCG-010	0.326	0.318	0.361
Butyricimonas	Odoribacter	0.377	0.398	0.385
Butyricimonas	Alistipes	0.407	0.366	0.414
Butyricimonas	NK4A214 group	0.327	0.326	0.330
Butyricimonas	UCG-002	0.313	0.440	0.391
Butyricimonas	UCG-005	0.361	0.452	0.388
Butyricimonas	UCG-010	0.357	0.376	0.324
Odoribacter	Alistipes	0.400	0.462	0.460
Odoribacter	Clostridia vadinBB60 group	0.320	0.346	0.312
Odoribacter	UCG-003	0.311	0.445	0.415
Odoribacter	UCG-005	0.334	0.453	0.423
Odoribacter	UCG-010	0.395	0.353	0.362
Alistipes	Parabacteroides	0.424	0.411	0.343
Alistipes	Christensenellaceae R-7 group	0.446	0.476	0.456
Alistipes	NK4A214 group	0.370	0.440	0.416
Alistipes	UCG-002	0.454	0.599	0.537
Alistipes	UCG-003	0.316	0.417	0.361
Alistipes	UCG-005	0.382	0.491	0.468
Alistipes	Eubacterium coprostanoligenes group	0.438	0.346	0.525
Erysipelatoclostridium	Clostridium innocuum group	0.432	0.560	0.409

Erysipelatoclostridium	Ruminococcus gnavus group	0.487	0.405	0.505
Erysipelatoclostridium	UCG-002	-0.346	-0.354	-0.321
Turicibacter	Intestinibacter	0.396	0.335	0.359
Clostridium innocuum group	Hungatella	0.302	0.356	0.367
Clostridium innocuum group	Ruminococcus gnavus group	0.326	0.326	0.423
RF39	Clostridia UCG-014	0.473	0.520	0.477
RF39	Eubacterium siraeum group	0.301	0.374	0.311
Christensenellaceae R-7 group	Clostridia UCG-014	0.365	0.552	0.519
Christensenellaceae R-7 group	Clostridia vadinBB60 group	0.331	0.393	0.358
Christensenellaceae R-7 group	Ruminococcus gnavus group	-0.348	-0.437	-0.434
Christensenellaceae R-7 group	NK4A214 group	0.470	0.627	0.584
Christensenellaceae R-7 group	UCG-002	0.556	0.729	0.682
Christensenellaceae R-7 group	UCG-005	0.544	0.733	0.608
Christensenellaceae R-7 group	Ruminococcus	0.361	0.479	0.401
Christensenellaceae R-7 group	Eubacterium siraeum group	0.312	0.459	0.373
Christensenellaceae R-7 group	UCG-010	0.451	0.527	0.499
Christensenellaceae R-7 group	Eubacterium coprostanoligenes group	0.489	0.559	0.595
Christensenellaceae R-7 group	Family XIII AD3011 group	0.345	0.343	0.428
Clostridia UCG-014	Clostridia vadinBB60 group	0.314	0.319	0.349
Clostridia UCG-014	Eubacterium ruminantium group	0.303	0.359	0.308
Clostridia UCG-014	Eubacterium xylanophilum group	0.303	0.317	0.325
Clostridia UCG-014	UCG-002	0.401	0.482	0.460
Clostridia UCG-014	Eubacterium siraeum group	0.311	0.361	0.391
Clostridia UCG-014	UCG-010	0.312	0.439	0.470
Clostridia UCG-014	Eubacterium coprostanoligenes group	0.316	0.387	0.372
Clostridia vadinBB60 group	Eubacterium xylanophilum group	0.376	0.334	0.405
Clostridia vadinBB60 group	NK4A214 group	0.351	0.342	0.365
Clostridia vadinBB60 group	UCG-005	0.394	0.438	0.430
Clostridia vadinBB60 group	Eubacterium siraeum group	0.432	0.346	0.366
Clostridia vadinBB60 group	UCG-010	0.542	0.513	0.537
Clostridium sensu stricto 1	Romboutsia	0.412	0.369	0.497
Anaerostipes	Eubacterium hallii group	0.343	0.329	0.460

Coprococcus	Dorea	0.473	0.417	0.421
Coprococcus	Lachnospiraceae NK4A136 group	0.475	0.483	0.398
Coprococcus	Roseburia	0.372	0.342	0.358
Coprococcus	Ruminococcus gauvreauii group	0.307	0.333	0.342
Coprococcus	UCG-002	0.379	0.482	0.419
Coprococcus	Eubacterium coprostanoligenes group	0.305	0.334	0.405
Dorea	Fusicatenibacter	0.414	0.352	0.413
Dorea	Eubacterium hallii group	0.323	0.405	0.363
Dorea	Ruminococcus torques group	0.348	0.341	0.331
Eisenbergiella	UBA1819	0.378	0.326	0.323
Fusicatenibacter	Eubacterium hallii group	0.360	0.386	0.302
Lachnoclostridium	Flavonifractor	0.346	0.313	0.394
Lachnospira	Lachnospiraceae UCG-001	0.422	0.358	0.440
Lachnospira	Lachnospiraceae UCG-004	0.401	0.354	0.378
Lachnospiraceae NK4A136 group	Lachnospiraceae UCG-001	0.338	0.410	0.363
Lachnospiraceae NK4A136 group	Roseburia	0.426	0.518	0.498
Lachnospiraceae NK4A136 group	Eubacterium eligens group	0.332	0.373	0.367
Lachnospiraceae NK4A136 group	Eubacterium xylanophilum group	0.304	0.328	0.348
Lachnospiraceae NK4A136 group	NK4A214 group	0.338	0.367	0.389
Lachnospiraceae NK4A136 group	UCG-002	0.424	0.511	0.481
Lachnospiraceae NK4A136 group	UCG-003	0.372	0.485	0.372
Lachnospiraceae NK4A136 group	Faecalibacterium	0.392	0.381	0.347
Lachnospiraceae NK4A136 group	Eubacterium siraeum group	0.337	0.341	0.333
Lachnospiraceae NK4A136 group	Eubacterium coprostanoligenes group	0.316	0.328	0.489
Lachnospiraceae UCG-001	Lachnospiraceae UCG-004	0.378	0.337	0.322
Lachnospiraceae UCG-001	Eubacterium eligens group	0.357	0.355	0.339
Lachnospiraceae UCG-001	Eubacterium xylanophilum group	0.367	0.467	0.508
Lachnospiraceae UCG-001	UCG-003	0.303	0.415	0.411
Lachnospiraceae UCG-004	UCG-003	0.312	0.303	0.334
Roseburia	Monoglobus	0.357	0.403	0.346
Roseburia	UCG-002	0.307	0.388	0.395
Roseburia	UCG-003	0.306	0.348	0.350

Roseburia	Faecalibacterium	0.411	0.318	0.378
Eubacterium xylanophilum group	UCG-003	0.312	0.406	0.436
Eubacterium xylanophilum group	Eubacterium siraeum group	0.400	0.428	0.405
Eubacterium xylanophilum group	UCG-010	0.362	0.430	0.444
Ruminococcus gnavus group	UCG-002	-0.455	-0.453	-0.457
Colidextribacter	Oscillibacter	0.369	0.353	0.387
Flavonifractor	UBA1819	0.320	0.373	0.339
NK4A214 group	UCG-002	0.541	0.659	0.594
NK4A214 group	UCG-003	0.456	0.563	0.459
NK4A214 group	UCG-005	0.530	0.689	0.617
NK4A214 group	Eubacterium siraeum group	0.328	0.319	0.358
NK4A214 group	UCG-010	0.429	0.566	0.502
NK4A214 group	Eubacterium coprostanoligenes group	0.419	0.530	0.474
Oscillibacter	UBA1819	0.342	0.350	0.354
UCG-002	UCG-003	0.426	0.657	0.467
UCG-002	UCG-005	0.519	0.771	0.639
UCG-002	Ruminococcus	0.461	0.420	0.380
UCG-002	Subdoligranulum	0.342	0.378	0.364
UCG-002	Eubacterium siraeum group	0.306	0.408	0.351
UCG-002	UCG-010	0.364	0.515	0.451
UCG-002	Eubacterium coprostanoligenes group	0.540	0.637	0.635
UCG-003	UCG-005	0.374	0.609	0.513
UCG-003	Eubacterium siraeum group	0.365	0.326	0.376
UCG-003	UCG-010	0.313	0.423	0.448
UCG-005	Ruminococcus	0.308	0.437	0.333
UCG-005	Eubacterium siraeum group	0.358	0.456	0.442
UCG-005	UCG-010	0.495	0.619	0.563
UCG-005	Eubacterium coprostanoligenes group	0.483	0.573	0.507
UCG-005	Family XIII AD3011 group	0.347	0.385	0.319
Incertae Sedis	UBA1819	0.370	0.421	0.341
Ruminococcus	Eubacterium coprostanoligenes group	0.464	0.460	0.477
Eubacterium siraeum group	UCG-010	0.429	0.435	0.428

UCG-010	Family XIII AD3011 group	0.303	0.303	0.335
Eubacterium coprostanoligenes group	Family XIII AD3011 group	0.357	0.351	0.325
Intestinibacter	Terrisporobacter	0.336	0.400	0.374
Veillonella	Haemophilus	0.311	0.399	0.329

Note: Bacterial genera nodes that were not annotated with a specific name were not displayed in the table. AD: Alzheimer's disease; MCI: Mild cognitive impairment; NC: Normal control.

Supplementary Table 2. Topological metrics of gut microbiota networks in the AD dementia, MCI, and NC groups

Groups	Node name	MCC	DMNC	Degree	EPC	Closeness	Radiality	Betweenness	Stress	ClusteringCoefficient
AD dementia	Christensenellaceae R-7 group	17	0.38	7	26.95	24.58	4.17	172.48	510	0.38
AD dementia	UCG-002	15	0.26	8	26.53	26.72	4.35	673.50	1666	0.25
AD dementia	UCG-005	14	0.38	10	27.43	28.08	4.44	790.21	1668	0.11
AD dementia	NK4A214 group	10	0.32	5	25.66	23.55	4.16	179.42	630	0.50
AD dementia	Faecalibacterium	9	0.31	8	24.22	25.27	4.16	745.25	1520	0.07
AD dementia	Lachnospiraceae NK4A136 group	8	0.31	6	23.36	23.15	4.01	373.00	1072	0.27
AD dementia	Alistipes	8	0.31	7	25.75	24.52	4.14	327.26	722	0.19
AD dementia	Clostridia UCG-014	7	0.28	5	25.84	23.72	4.17	99.16	286	0.30
AD dementia	Eubacterium coprostanoligenes group	7	0.31	7	25.87	26.08	4.37	612.26	1106	0.05
AD dementia	UCG-010	7	0.31	6	26.00	23.07	4.08	124.70	386	0.20
AD dementia	Clostridia vadinBB60 group	6	0.31	5	22.34	21.53	3.91	227.83	488	0.20
AD dementia	Oscillibacter	6	0.31	5	21.86	21.35	3.79	272.90	538	0.20
AD dementia	UBA1819	5	0.00	5	18.09	21.30	3.84	393.36	616	0.00
AD dementia	Fusicatenibacter	5	0.00	5	15.45	19.89	3.61	347.63	660	0.00
AD dementia	Coprococcus	5	0.00	5	21.55	24.00	4.21	565.44	998	0.00
AD dementia	Eubacterium eligens group	4	0.31	4	16.33	18.92	3.51	153.13	386	0.17
AD dementia	Lachnospiraceae UCG-001	4	0.31	3	16.84	18.04	3.47	10.33	26	0.67
AD dementia	Roseburia	4	0.00	4	14.85	19.58	3.70	269.58	436	0.00
AD dementia	Eggerthella	4	0.00	4	18.68	20.92	3.90	274.39	572	0.00
AD dementia	Rothia	4	0.00	4	14.43	21.17	3.93	610.91	920	0.00
AD dementia	Lachnospira	3	0.31	3	16.93	18.04	3.46	27.93	72	0.33
AD dementia	Family XIII AD3011 group	3	0.00	3	22.02	21.27	3.98	11.62	30	0.00
AD dementia	Colidextribacter	3	0.31	3	21.10	20.48	3.86	20.75	28	0.33
AD dementia	Barnesiella	3	0.31	3	21.77	21.08	3.94	7.92	20	0.33
AD dementia	Bacteroides	3	0.31	3	17.95	18.85	3.61	54.65	146	0.33
AD dementia	Veillonella	3	0.00	3	7.67	16.63	3.32	226.00	338	0.00
AD dementia	Erysipelatoclostridium	3	0.00	3	16.30	20.16	3.85	226.20	364	0.00
AD dementia	Subdoligranulum	2	0.31	2	18.26	19.82	3.85	0.00	0	1.00
AD dementia	Lachnoclostridium	2	0.00	2	10.65	15.83	3.16	114.00	224	0.00
AD dementia	Erysipelotrichaceae UCG-003	2	0.00	2	14.03	17.73	3.53	16.37	46	0.00

AD dementia	Dorea	2	0.00	2	12.69	17.42	3.57	15.92	46	0.00
AD dementia	Ruminococcus	2	0.31	2	18.11	19.42	3.84	0.00	0	1.00
AD dementia	Odoribacter	2	0.00	2	14.33	18.58	3.70	65.02	168	0.00
AD dementia	Parabacteroides	2	0.00	2	13.10	15.70	3.19	9.67	26	0.00
AD dementia	UCG-003	2	0.00	2	17.26	20.92	4.03	43.43	74	0.00
AD dementia	Ruminococcus gnavus group	2	0.00	2	15.56	19.60	3.88	61.06	148	0.00
AD dementia	Clostridium innocuum group	2	0.00	2	9.11	15.81	3.26	114.00	220	0.00
AD dementia	Granulicatella	2	0.00	2	7.91	15.96	3.29	114.00	170	0.00
AD dementia	Actinomyces	2	0.00	2	11.29	18.32	3.75	170.71	240	0.00
AD dementia	Klebsiella	1	0.00	1	1.41	1.00	0.07	0.00	0	0.00
AD dementia	Enterobacter	1	0.00	1	1.41	1.00	0.07	0.00	0	0.00
AD dementia	Haemophilus	1	0.00	1	4.18	12.75	2.66	0.00	0	0.00
AD dementia	Lachnospiraceae UCG-004	1	0.00	1	7.98	13.92	2.86	0.00	0	0.00
AD dementia	Incertae Sedis	1	0.00	1	8.69	15.20	3.18	0.00	0	0.00
AD dementia	Flavonifractor	1	0.00	1	8.41	15.20	3.18	0.00	0	0.00
AD dementia	Butyricicoccus	1	0.00	1	7.27	14.36	3.04	0.00	0	0.00
AD dementia	Monoglobus	1	0.00	1	11.36	17.06	3.50	0.00	0	0.00
AD dementia	Eubacterium ventriosum group	1	0.00	1	7.62	14.36	3.04	0.00	0	0.00
AD dementia	Lachnospiraceae FCS020 group	1	0.00	1	7.57	14.35	2.95	0.00	0	0.00
AD dementia	Eubacterium hallii group	1	0.00	1	7.42	14.35	2.95	0.00	0	0.00
AD dementia	Anaerostipes	1	0.00	1	7.79	14.35	2.95	0.00	0	0.00
AD dementia	Eisenbergiella	1	0.00	1	8.33	15.20	3.18	0.00	0	0.00
AD dementia	Eubacterium xylanophilum group	1	0.00	1	10.99	15.35	3.25	0.00	0	0.00
AD dementia	Eubacterium siraeum group	1	0.00	1	12.21	18.45	3.78	0.00	0	0.00
AD dementia	Gemella	1	0.00	1	4.40	12.50	2.64	0.00	0	0.00
AD dementia	Hungatella	1	0.00	1	5.01	12.40	2.60	0.00	0	0.00
AD dementia	Intestinibacter	1	0.00	1	1.44	1.00	0.07	0.00	0	0.00
AD dementia	Turicibacter	1	0.00	1	1.44	1.00	0.07	0.00	0	0.00
AD dementia	Streptococcus	1	0.00	1	4.13	12.75	2.66	0.00	0	0.00
AD dementia	Anaerococcus	0	0.00	0	1.00	0.00	0.00	0.00	0	0.00
AD dementia	Anaerotruncus	0	0.00	0	1.00	0.00	0.00	0.00	0	0.00
AD dementia	Tyzzerella	0	0.00	0	1.00	0.00	0.00	0.00	0	0.00

AD dementia	Sellimonas	0	0.00	0	1.00	0.00	0.00	0.00	0	0.00
AD dementia	Ruminococcus torques group	0	0.00	0	1.00	0.00	0.00	0.00	0	0.00
AD dementia	Ruminococcus gauvreauii group	0	0.00	0	1.00	0.00	0.00	0.00	0	0.00
AD dementia	Blautia	0	0.00	0	1.00	0.00	0.00	0.00	0	0.00
AD dementia	Eubacterium	0	0.00	0	1.00	0.00	0.00	0.00	0	0.00
AD dementia	Romboutsia	0	0.00	0	1.00	0.00	0.00	0.00	0	0.00
AD dementia	Eubacterium ruminantium group	0	0.00	0	1.00	0.00	0.00	0.00	0	0.00
AD dementia	RF39	0	0.00	0	1.00	0.00	0.00	0.00	0	0.00
AD dementia	Terrisporobacter	0	0.00	0	1.00	0.00	0.00	0.00	0	0.00
AD dementia	Clostridium sensu stricto 1	0	0.00	0	1.00	0.00	0.00	0.00	0	0.00
AD dementia	Enterococcus	0	0.00	0	1.00	0.00	0.00	0.00	0	0.00
AD dementia	Holdemanella	0	0.00	0	1.00	0.00	0.00	0.00	0	0.00
AD dementia	Catenibacterium	0	0.00	0	1.00	0.00	0.00	0.00	0	0.00
AD dementia	Desulfovibrio	0	0.00	0	1.00	0.00	0.00	0.00	0	0.00
AD dementia	Bilophila	0	0.00	0	1.00	0.00	0.00	0.00	0	0.00
AD dementia	Phascolarctobacterium	0	0.00	0	1.00	0.00	0.00	0.00	0	0.00
AD dementia	Peptoniphilus	0	0.00	0	1.00	0.00	0.00	0.00	0	0.00
AD dementia	Porphyromonas	0	0.00	0	1.00	0.00	0.00	0.00	0	0.00
AD dementia	Butyricimonas	0	0.00	0	1.00	0.00	0.00	0.00	0	0.00
AD dementia	Chloroplast	0	0.00	0	1.00	0.00	0.00	0.00	0	0.00
AD dementia	Atopobium	0	0.00	0	1.00	0.00	0.00	0.00	0	0.00
AD dementia	Methanobrevibacter	0	0.00	0	1.00	0.00	0.00	0.00	0	0.00
MCI	UCG-005	78	0.23	18	22.89	39.17	3.41	676.91	2202	0.20
MCI	UCG-002	72	0.23	19	22.41	38.92	3.37	814.30	2342	0.16
MCI	UCG-003	34	0.24	15	21.38	35.83	3.24	561.27	1726	0.13
MCI	Christensenellaceae R-7 group	31	0.29	10	19.54	32.00	3.10	107.77	294	0.27
MCI	RF39	30	0.40	7	18.15	30.83	3.11	29.56	114	0.52
MCI	UCG-010	30	0.31	11	20.21	32.33	3.10	215.80	782	0.24
MCI	Flavonifractor	29	0.37	12	18.91	35.08	3.28	802.04	1908	0.17
MCI	Eubacterium siraeum group	24	0.26	9	19.67	32.58	3.18	66.47	344	0.31
MCI	Ruminococcus gnavus group	22	0.33	9	17.88	32.67	3.19	240.31	622	0.25
MCI	Odoribacter	20	0.33	7	18.64	31.08	3.12	70.45	312	0.43

MCI	Coprococcus	18	0.47	10	17.34	32.92	3.16	629.91	1598	0.11
MCI	Clostridia UCG-014	17	0.33	7	17.21	29.92	3.04	24.97	120	0.33
MCI	NK4A214 group	16	0.26	9	18.56	31.92	3.13	141.73	516	0.19
MCI	Eubacterium ruminantium group	14	0.26	7	17.58	30.50	3.07	87.94	470	0.33
MCI	Subdoligranulum	13	0.47	5	14.93	30.25	3.12	282.07	578	0.50
MCI	Eubacterium coprostanoligenes group	13	0.24	9	18.49	33.08	3.21	271.80	926	0.17
MCI	Barnesiella	13	0.47	5	14.57	28.25	2.95	128.50	302	0.50
MCI	Eubacterium xylanophilum group	11	0.32	6	15.55	29.08	3.00	42.29	166	0.33
MCI	Eggerthella	10	0.38	6	15.60	28.83	2.98	49.57	238	0.27
MCI	Lachnospiraceae UCG-001	9	0.28	7	14.64	30.42	3.06	231.23	632	0.14
MCI	GCA-900066575	8	0.28	6	14.38	29.50	3.02	158.68	512	0.20
MCI	Lachnospiraceae NK4A136 group	8	0.31	7	15.48	29.85	3.00	217.00	650	0.10
MCI	Alistipes	7	0.31	7	15.35	30.83	3.10	246.69	758	0.10
MCI	Clostridium innocuum group	7	0.31	7	11.21	28.28	2.89	338.08	858	0.05
MCI	Prevotella	7	0.31	6	12.12	27.62	2.86	378.50	1262	0.13
MCI	Ruminococcus torques group	6	0.28	4	14.42	29.00	3.05	20.65	148	0.50
MCI	Erysipelatoclostridium	6	0.31	6	14.67	31.00	3.14	210.89	578	0.13
MCI	Clostridia vadinBB60 group	5	0.31	5	14.23	28.17	2.94	43.27	224	0.10
MCI	Lachnospiraceae UCG-004	4	0.31	3	6.77	22.43	2.52	4.80	24	0.67
MCI	Eubacterium hallii group	4	0.31	4	6.67	23.90	2.60	228.51	532	0.17
MCI	Family XIII UCG-001	4	0.31	3	11.93	26.58	2.89	1.17	12	0.67
MCI	Fusicatenibacter	4	0.31	4	5.93	23.58	2.64	215.48	432	0.17
MCI	UBA1819	4	0.31	4	7.18	25.28	2.75	254.00	498	0.17
MCI	Family XIII AD3011 group	3	0.31	3	10.26	26.03	2.83	46.47	126	0.33
MCI	Ruminococcus	3	0.00	3	12.56	27.83	2.99	6.97	70	0.00
MCI	Butyricimonas	3	0.00	3	8.54	24.87	2.71	30.17	72	0.00
MCI	Chloroplast	3	0.00	3	10.04	25.53	2.79	31.82	104	0.00
MCI	Lachnospira	2	0.31	2	4.79	20.97	2.42	0.00	0	1.00
MCI	Ruminococcus gauvreauii group	2	0.00	2	5.74	22.58	2.58	27.58	82	0.00
MCI	Lachnospiraceae FCS020 group	2	0.00	2	8.08	24.23	2.73	6.09	22	0.00
MCI	Lachnoclostridium	2	0.00	2	8.16	24.42	2.76	2.50	12	0.00
MCI	Dorea	2	0.31	2	3.40	18.87	2.14	0.00	0	1.00

MCI	TM7x	2	0.00	2	5.98	21.53	2.48	3.60	12	0.00
MCI	Colidextribacter	2	0.00	2	5.99	21.92	2.52	4.97	20	0.00
MCI	Tyzzerella	2	0.00	2	6.81	23.75	2.71	12.20	42	0.00
MCI	Marvinbryantia	2	0.00	2	5.08	21.58	2.49	17.59	44	0.00
MCI	Veillonella	1	0.00	1	4.63	20.73	2.43	0.00	0	0.00
MCI	Incertae Sedis	1	0.00	1	2.75	18.07	2.10	0.00	0	0.00
MCI	Fusobacterium	1	0.00	1	5.23	22.45	2.63	0.00	0	0.00
MCI	Faecalibacterium	1	0.00	1	1.21	1.00	0.06	0.00	0	0.00
MCI	Eubacterium eligens group	1	0.00	1	5.80	22.53	2.59	0.00	0	0.00
MCI	Roseburia	1	0.00	1	4.33	21.48	2.51	0.00	0	0.00
MCI	Granulicatella	1	0.00	1	1.19	1.00	0.06	0.00	0	0.00
MCI	Izemoplasmatales	1	0.00	1	4.90	21.10	2.45	0.00	0	0.00
MCI	Intestinibacter	1	0.00	1	1.19	1.00	0.06	0.00	0	0.00
MCI	Turicibacter	1	0.00	1	1.19	1.00	0.06	0.00	0	0.00
MCI	Butyricicoccus	1	0.00	1	1.21	1.00	0.06	0.00	0	0.00
MCI	Erysipelotrichaceae UCG-003	1	0.00	1	2.30	17.20	1.99	0.00	0	0.00
MCI	Eisenbergiella	1	0.00	1	3.02	19.35	2.24	0.00	0	0.00
MCI	CAG-56	1	0.00	1	4.28	20.88	2.49	0.00	0	0.00
MCI	Candidatus Stoquefichus	1	0.00	1	3.37	19.35	2.24	0.00	0	0.00
MCI	Oscillibacter	1	0.00	1	2.55	18.07	2.10	0.00	0	0.00
MCI	Dialister	1	0.00	1	3.82	19.08	2.21	0.00	0	0.00
MCI	Holdemanella	1	0.00	1	3.44	19.08	2.21	0.00	0	0.00
MCI	Eubacterium ventriosum group	1	0.00	1	3.98	19.53	2.30	0.00	0	0.00
MCI	Hungatella	1	0.00	1	4.83	22.45	2.63	0.00	0	0.00
MCI	Bifidobacterium	1	0.00	1	3.62	19.08	2.21	0.00	0	0.00
MCI	Streptococcus	1	0.00	1	1.19	1.00	0.06	0.00	0	0.00
MCI	Haemophilus	0	0.00	0	1.00	0.00	0.00	0.00	0	0.00
MCI	Phascolarctobacterium	0	0.00	0	1.00	0.00	0.00	0.00	0	0.00
MCI	Terrisporobacter	0	0.00	0	1.00	0.00	0.00	0.00	0	0.00
MCI	Anaerotruncus	0	0.00	0	1.00	0.00	0.00	0.00	0	0.00
MCI	Escherichia-Shigella	0	0.00	0	1.00	0.00	0.00	0.00	0	0.00
MCI	Monoglobus	0	0.00	0	1.00	0.00	0.00	0.00	0	0.00

MCI	Sellimonas	0	0.00	0	1.00	0.00	0.00	0.00	0	0.00
MCI	Anaerostipes	0	0.00	0	1.00	0.00	0.00	0.00	0	0.00
MCI	Romboutsia	0	0.00	0	1.00	0.00	0.00	0.00	0	0.00
MCI	Clostridium sensu stricto 1	0	0.00	0	1.00	0.00	0.00	0.00	0	0.00
MCI	Defluviitaleaceae UCG-011	0	0.00	0	1.00	0.00	0.00	0.00	0	0.00
MCI	Akkermansia	0	0.00	0	1.00	0.00	0.00	0.00	0	0.00
MCI	Gemella	0	0.00	0	1.00	0.00	0.00	0.00	0	0.00
MCI	Holdemania	0	0.00	0	1.00	0.00	0.00	0.00	0	0.00
MCI	Mitochondria	0	0.00	0	1.00	0.00	0.00	0.00	0	0.00
MCI	Bilophila	0	0.00	0	1.00	0.00	0.00	0.00	0	0.00
MCI	Muribaculaceae	0	0.00	0	1.00	0.00	0.00	0.00	0	0.00
MCI	Parabacteroides	0	0.00	0	1.00	0.00	0.00	0.00	0	0.00
MCI	Bacteroides	0	0.00	0	1.00	0.00	0.00	0.00	0	0.00
MCI	Blautia	0	0.00	0	1.00	0.00	0.00	0.00	0	0.00
MCI	Gordonibacter	0	0.00	0	1.00	0.00	0.00	0.00	0	0.00
MCI	Negativibacillus	0	0.00	0	1.00	0.00	0.00	0.00	0	0.00
MCI	Adlercreutzia	0	0.00	0	1.00	0.00	0.00	0.00	0	0.00
MCI	Rothia	0	0.00	0	1.00	0.00	0.00	0.00	0	0.00
MCI	Eubacterium brachy group	0	0.00	0	1.00	0.00	0.00	0.00	0	0.00
MCI	Actinomyces	0	0.00	0	1.00	0.00	0.00	0.00	0	0.00
MCI	CAG-352	0	0.00	0	1.00	0.00	0.00	0.00	0	0.00
MCI	Methanobrevibacter	0	0.00	0	1.00	0.00	0.00	0.00	0	0.00
NC	Christensenellaceae R-7 group	72	0.32	15	32.29	32.12	4.58	506.39	1226	0.24
NC	NK4A214 group	42	0.32	12	32.01	30.57	4.52	384.06	1026	0.24
NC	Eubacterium coprostanoligenes group	33	0.35	9	31.53	28.28	4.44	196.50	502	0.33
NC	UCG-002	28	0.37	9	31.04	28.07	4.41	448.67	1216	0.28
NC	Alistipes	25	0.43	7	30.73	26.62	4.37	76.14	292	0.43
NC	Eubacterium siraeum group	22	0.37	7	30.88	26.43	4.31	60.16	224	0.48
NC	UCG-005	22	0.37	9	31.15	27.57	4.37	257.66	624	0.28
NC	UCG-003	22	0.29	12	31.25	30.58	4.56	911.65	2122	0.15
NC	UCG-010	20	0.33	9	31.05	27.98	4.41	303.53	800	0.25
NC	Lachnospiraceae NK4A136 group	9	0.28	7	29.45	27.87	4.47	342.48	904	0.14

NC	Odoribacter	9	0.38	5	28.54	23.93	4.17	18.48	76	0.40
NC	Lachnospiraceae UCG-001	8	0.26	5	27.22	24.57	4.26	102.88	326	0.40
NC	Barnesiella	8	0.38	4	26.66	23.85	4.19	8.86	50	0.67
NC	Ruminococcus	7	0.46	4	26.18	23.40	4.16	35.40	120	0.50
NC	Roseburia	7	0.31	7	25.12	24.02	4.10	327.21	894	0.10
NC	Clostridia vadinBB60 group	7	0.46	4	24.07	23.43	4.16	112.00	202	0.50
NC	Ruminococcus gnavus group	7	0.31	6	26.82	26.15	4.35	321.99	658	0.13
NC	Lachnospiraceae UCG-004	6	0.28	4	24.10	22.55	4.09	13.18	42	0.50
NC	Family XIII AD3011 group	6	0.46	3	23.54	22.85	4.14	0.00	0	1.00
NC	Flavonifractor	6	0.31	5	20.22	21.04	3.87	112.61	276	0.30
NC	Eubacterium xylanophilum group	6	0.31	6	27.17	24.23	4.17	106.00	334	0.13
NC	Lachnospira	5	0.31	4	23.69	22.98	4.16	55.99	112	0.33
NC	Dorea	5	0.31	5	20.40	21.18	3.83	163.12	464	0.10
NC	Coprococcus	5	0.31	5	25.28	23.57	4.16	202.71	564	0.10
NC	Subdoligranulum	4	0.31	4	20.71	21.38	3.93	72.66	212	0.17
NC	Hungatella	4	0.31	3	13.66	17.74	3.48	10.27	30	0.67
NC	Clostridium innocuum group	4	0.31	3	14.05	17.74	3.48	10.27	30	0.67
NC	Erysipelatoclostridium	4	0.31	4	16.76	20.91	3.89	233.09	582	0.17
NC	Faecalibacterium	3	0.00	3	21.86	22.21	4.06	82.47	254	0.00
NC	Eubacterium hallii group	3	0.00	3	16.01	20.43	3.89	122.50	270	0.00
NC	Eubacterium ventriosum group	3	0.00	3	22.62	21.88	4.01	59.45	186	0.00
NC	Clostridium sensu stricto 1	3	0.00	3	2.24	3.50	0.21	10.00	10	0.00
NC	Rothia	3	0.00	3	5.82	16.88	3.42	328.00	670	0.00
NC	Incertae Sedis	2	0.00	2	14.93	19.24	3.80	17.20	52	0.00
NC	Eubacterium eligens group	2	0.00	2	15.85	18.57	3.73	2.07	6	0.00
NC	Butyricicoccus	2	0.31	2	12.62	16.99	3.47	0.00	0	1.00
NC	Monoglobus	2	0.31	2	12.85	16.99	3.47	0.00	0	1.00
NC	Ruminococcus gauvreauii group	2	0.31	2	14.26	18.01	3.64	0.00	0	1.00
NC	Lachnoclostridium	2	0.31	2	15.92	18.71	3.76	0.00	0	1.00
NC	Romboutsia	2	0.00	2	2.02	3.00	0.20	6.00	6	0.00
NC	Clostridia UCG-014	2	0.31	2	19.56	21.35	4.05	0.00	0	1.00
NC	Butyricimonas	2	0.31	2	19.93	21.35	4.06	0.00	0	1.00

NC	Colidextribacter	2	0.00	2	12.75	20.80	4.00	424.00	880	0.00
NC	Actinomyces	2	0.00	2	3.46	13.59	2.80	112.00	226	0.00
NC	Dialister	1	0.00	1	1.40	1.00	0.07	0.00	0	0.00
NC	Phascolarctobacterium	1	0.00	1	1.40	1.00	0.07	0.00	0	0.00
NC	Megasphaera	1	0.00	1	1.34	1.00	0.07	0.00	0	0.00
NC	Acidaminococcus	1	0.00	1	1.34	1.00	0.07	0.00	0	0.00
NC	Negativibacillus	1	0.00	1	11.51	19.10	3.84	0.00	0	0.00
NC	Family XIII UCG-001	1	0.00	1	12.39	18.40	3.76	0.00	0	0.00
NC	Ruminococcus torques group	1	0.00	1	8.13	15.09	3.18	0.00	0	0.00
NC	Fusicatenibacter	1	0.00	1	6.93	14.96	3.24	0.00	0	0.00
NC	Eubacterium ruminantium group	1	0.00	1	12.32	19.46	3.87	0.00	0	0.00
NC	Akkermansia	1	0.00	1	13.26	20.00	3.93	0.00	0	0.00
NC	Marvinbryantia	1	0.00	1	12.66	20.00	3.93	0.00	0	0.00
NC	RF39	1	0.00	1	11.82	18.40	3.76	0.00	0	0.00
NC	Terrisporobacter	1	0.00	1	1.71	2.33	0.17	0.00	0	0.00
NC	Intestinibacter	1	0.00	1	1.67	2.33	0.17	0.00	0	0.00
NC	Turicibacter	1	0.00	1	1.61	2.17	0.16	0.00	0	0.00
NC	Butyrivibrio	1	0.00	1	9.62	16.49	3.52	0.00	0	0.00
NC	Oscillibacter	1	0.00	1	12.85	19.10	3.84	0.00	0	0.00
NC	Sutterella	1	0.00	1	12.84	19.50	3.91	0.00	0	0.00
NC	Parabacteroides	1	0.00	1	1.40	1.00	0.07	0.00	0	0.00
NC	Bacteroides	1	0.00	1	1.40	1.00	0.07	0.00	0	0.00
NC	Eggerthella	1	0.00	1	7.11	15.11	3.24	0.00	0	0.00
NC	Chloroplast	1	0.00	1	2.98	12.92	2.78	0.00	0	0.00
NC	Streptococcus	1	0.00	1	2.00	10.95	2.15	0.00	0	0.00
NC	Klebsiella	0	0.00	0	1.00	0.00	0.00	0.00	0	0.00
NC	Enterobacter	0	0.00	0	1.00	0.00	0.00	0.00	0	0.00
NC	Haemophilus	0	0.00	0	1.00	0.00	0.00	0.00	0	0.00
NC	UBA1819	0	0.00	0	1.00	0.00	0.00	0.00	0	0.00
NC	Eisenbergiella	0	0.00	0	1.00	0.00	0.00	0.00	0	0.00
NC	Blautia	0	0.00	0	1.00	0.00	0.00	0.00	0	0.00
NC	Anaerostipes	0	0.00	0	1.00	0.00	0.00	0.00	0	0.00

NC	Veillonella	0	0.00	0	1.00	0.00	0.00	0.00	0	0.00
NC	Lactobacillus	0	0.00	0	1.00	0.00	0.00	0.00	0	0.00
NC	Izemoplasmatales	0	0.00	0	1.00	0.00	0.00	0.00	0	0.00
NC	Holdemanella	0	0.00	0	1.00	0.00	0.00	0.00	0	0.00
NC	Prevotella	0	0.00	0	1.00	0.00	0.00	0.00	0	0.00
NC	Catenibacterium	0	0.00	0	1.00	0.00	0.00	0.00	0	0.00
NC	Rikenellaceae RC9 gut group	0	0.00	0	1.00	0.00	0.00	0.00	0	0.00
NC	Alloprevotella	0	0.00	0	1.00	0.00	0.00	0.00	0	0.00
NC	Granulicatella	0	0.00	0	1.00	0.00	0.00	0.00	0	0.00
NC	Atopobium	0	0.00	0	1.00	0.00	0.00	0.00	0	0.00

Note: Bacterial genera nodes that were not annotated with a specific name were not displayed in the table. MCC: Maximal clique centrality; DMNC: Density of maximum neighborhood component; EPC: Edge percolated component; AD: Alzheimer's disease; MCI: Mild cognitive impairment; NC: Normal control.

Supplementary Table 3. Comparison of candidate gut microbiota indicator differences between groups

Candidate indicator	Comparison	Z-values	*P*-values
Methanobrevibacter	AD dementia vs MCI	3.32	0.001
Methanobrevibacter	AD dementia vs NC	4.53	<0.001
Methanobrevibacter	MCI vs NC	-0.57	0.570
Olsenella	AD dementia vs MCI	3.64	<0.001
Olsenella	AD dementia vs NC	3.71	0.001
Olsenella	MCI vs NC	-1.38	0.169
Collinsella	AD dementia vs MCI	4.62	<0.001
Collinsella	AD dementia vs NC	4.46	<0.001
Collinsella	MCI vs NC	-1.90	0.057
Bacteroides	AD dementia vs MCI	-4.32	<0.001
Bacteroides	AD dementia vs NC	-5.86	<0.001
Bacteroides	MCI vs NC	0.76	0.445
Alistipes	AD dementia vs MCI	-4.47	<0.001
Alistipes	AD dementia vs NC	-3.45	0.001
Alistipes	MCI vs NC	2.36	0.018
Parabacteroides	AD dementia vs MCI	-2.37	0.027
Parabacteroides	AD dementia vs NC	-4.06	<0.001
Parabacteroides	MCI vs NC	-0.09	0.930
Erysipelatoclostridium	AD dementia vs MCI	2.78	0.016
Erysipelatoclostridium	AD dementia vs NC	2.60	0.014
Erysipelatoclostridium	MCI vs NC	-1.19	0.232
Enterococcus	AD dementia vs MCI	4.95	<0.001
Enterococcus	AD dementia vs NC	5.17	<0.001
Enterococcus	MCI vs NC	-1.80	0.072
Lactobacillus	AD dementia vs MCI	4.26	<0.001
Lactobacillus	AD dementia vs NC	6.63	<0.001
Lactobacillus	MCI vs NC	-0.24	0.808
Lactococcus	AD dementia vs MCI	2.44	0.022
Lactococcus	AD dementia vs NC	-1.77	0.077

Lactococcus	MCI vs NC	-3.48	0.002
RF39	AD dementia vs MCI	-2.57	0.015
RF39	AD dementia vs NC	-3.42	0.002
RF39	MCI vs NC	0.49	0.626
Clostridium sensu stricto 1	AD dementia vs MCI	2.00	0.069
Clostridium sensu stricto 1	AD dementia vs NC	-1.74	0.082
Clostridium sensu stricto 1	MCI vs NC	-3.03	0.007
Eubacterium	AD dementia vs MCI	2.98	0.004
Eubacterium	AD dementia vs NC	4.19	<0.001
Eubacterium	MCI vs NC	-0.44	0.664
Coprococcus	AD dementia vs MCI	-3.64	<0.001
Coprococcus	AD dementia vs NC	-5.75	<0.001
Coprococcus	MCI vs NC	0.16	0.873
Eisenbergiella	AD dementia vs MCI	2.88	0.006
Eisenbergiella	AD dementia vs NC	3.34	0.003
Eisenbergiella	MCI vs NC	-0.85	0.394
Fusicatenibacter	AD dementia vs MCI	-6.08	<0.001
Fusicatenibacter	AD dementia vs NC	-5.16	<0.001
Fusicatenibacter	MCI vs NC	2.93	0.003
Lachnospira	AD dementia vs MCI	-4.48	<0.001
Lachnospira	AD dementia vs NC	-7.27	<0.001
Lachnospira	MCI vs NC	0.07	0.941
Lachnospiraceae UCG-001	AD dementia vs MCI	-2.38	0.026
Lachnospiraceae UCG-001	AD dementia vs NC	-6.29	<0.001
Lachnospiraceae UCG-001	MCI vs NC	-1.42	0.156
Roseburia	AD dementia vs MCI	-5.83	<0.001
Roseburia	AD dementia vs NC	-5.84	<0.001
Roseburia	MCI vs NC	2.27	0.023
Sellimonas	AD dementia vs MCI	2.84	0.013
Sellimonas	AD dementia vs NC	2.46	0.021
Sellimonas	MCI vs NC	-1.34	0.179
Eubacterium eligens group	AD dementia vs MCI	-4.04	<0.001

Eubacterium eligens group	AD dementia vs NC	-4.93	<0.001
Eubacterium eligens group	MCI vs NC	1.05	0.295
Eubacterium ventriosum group	AD dementia vs MCI	-4.47	<0.001
Eubacterium ventriosum group	AD dementia vs NC	-4.82	<0.001
Eubacterium ventriosum group	MCI vs NC	1.54	0.125
Butyricicoccus	AD dementia vs MCI	-3.89	<0.001
Butyricicoccus	AD dementia vs NC	-7.19	<0.001
Butyricicoccus	MCI vs NC	-0.46	0.643
UCG-003	AD dementia vs MCI	-4.40	<0.001
UCG-003	AD dementia vs NC	-3.10	0.003
UCG-003	MCI vs NC	2.51	0.012
Faecalibacterium	AD dementia vs MCI	-6.73	<0.001
Faecalibacterium	AD dementia vs NC	-6.52	<0.001
Faecalibacterium	MCI vs NC	2.76	0.006
Romboutsia	AD dementia vs MCI	-0.15	0.879
Romboutsia	AD dementia vs NC	-5.01	<0.001
Romboutsia	MCI vs NC	-2.86	0.006
Peptoniphilus	AD dementia vs MCI	3.39	0.001
Peptoniphilus	AD dementia vs NC	5.09	<0.001
Peptoniphilus	MCI vs NC	-0.30	0.765
Phascolarctobacterium	AD dementia vs MCI	-2.89	0.011
Phascolarctobacterium	AD dementia vs NC	-2.14	0.048
Phascolarctobacterium	MCI vs NC	1.58	0.113
Dialister	AD dementia vs MCI	-5.17	<0.001
Dialister	AD dementia vs NC	-6.43	<0.001
Dialister	MCI vs NC	1.27	0.205
Parasutterella	AD dementia vs MCI	-4.69	<0.001
Parasutterella	AD dementia vs NC	-6.54	<0.001
Parasutterella	MCI vs NC	0.72	0.470

Sutterella	AD dementia vs MCI	-4.63	<0.001
Sutterella	AD dementia vs NC	-3.19	0.002
Sutterella	MCI vs NC	2.68	0.007
Enterobacter	AD dementia vs MCI	4.73	<0.001
Enterobacter	AD dementia vs NC	3.16	0.002
Enterobacter	MCI vs NC	-2.79	0.005
Acinetobacter	AD dementia vs MCI	4.89	<0.001
Acinetobacter	AD dementia vs NC	2.31	0.021
Acinetobacter	MCI vs NC	-3.46	0.001
Akkermansia	AD dementia vs MCI	6.62	<0.001
Akkermansia	AD dementia vs NC	6.29	<0.001
Akkermansia	MCI vs NC	-2.79	0.005
Oligella	AD dementia vs MCI	2.56	0.016
Oligella	AD dementia vs NC	4.89	<0.001
Oligella	MCI vs NC	0.40	0.687

Note: *P*-values were derived from Dunn's test, and Benjamini-Hochberg correction was applied.

AD: Alzheimer's disease; MCI: Mild cognitive impairment; NC: Normal control.

Supplementary Table 4. Ranking of variable importance in different models in the overall population

Variable	RF	GBM	XGBoost	Average value
Faecalibacterium	1.00	2.00	1.00	1.33
Akkermansia	2.00	6.00	2.00	3.33
Bacteroides	3.00	1.00	8.00	4.00
Parabacteroides	4.00	7.00	3.00	4.67
Fusicatenibacter	8.00	4.00	5.00	5.67
Roseburia	10.00	3.00	7.00	6.67
Collinsella	6.00	9.00	6.00	7.00
Dialister	17.00	5.00	4.00	8.67
Romboutsia	7.00	10.00	11.00	9.33
Butyricicoccus	11.00	8.00	12.00	10.33
Clostridium sensu stricto 1	5.00	19.00	16.00	13.33
Coprococcus	13.00	13.00	14.00	13.33
Parasutterella	20.00	12.00	9.00	13.67
Sutterella	21.00	11.00	10.00	14.00
Alistipes	12.00	17.00	15.00	14.67
Phascolarctobacterium	15.00	15.00	17.00	15.67
Lactobacillus	14.00	21.00	13.00	16.00
Enterobacter	9.00	20.00	21.00	16.67
Eubacterium ventriosum group	19.00	18.00	18.00	18.33
Lachnospira	22.00	14.00	20.00	18.67
Eubacterium eligens group	23.00	16.00	22.00	20.33
UCG-003	25.00	22.00	19.00	22.00
Erysipelatoclostridium	18.00	26.00	25.00	23.00
Acinetobacter	16.00	29.00	26.00	23.67
RF39	28.00	24.00	23.00	25.00
Lachnospiraceae UCG-001	26.00	25.00	27.00	26.00
Lactococcus	27.00	23.00	28.00	26.00
Enterococcus	24.00	32.00	24.00	26.67
Eisenbergiella	29.00	28.00	29.00	28.67

Peptoniphilus	33.00	27.00	30.00	30.00
Sellimonas	30.00	31.00	33.00	31.33
Methanobrevibacter	34.00	30.00	31.00	31.67
Eubacterium	32.00	33.00	32.00	32.33
Olsenella	31.00	34.00	34.00	33.00
Oligella	35.00	35.00	35.00	35.00

RF: Random Forest; GBM: Gradient Boosting Machine; XGBoost: Extreme Gradient Boosting.

Supplementary Table 5. Performance of different discriminative models on the test set under sensitivity analysis of candidate indicators

Groups	Model	Sensitivity	Specificity	PPV	NPV	Accuracy	F1 score	AUC(95% CI)
Overall population (N=1700)	RF	0.747	0.707	0.540	0.848	0.713	0.579	0.791 (0.772 ~ 0.810)
Overall population (N=1700)	GBM	0.699	0.715	0.522	0.817	0.705	0.56	0.750 (0.729 ~ 0.772)
Overall population (N=1700)	XGBoost	0.754	0.689	0.527	0.83	0.686	0.564	0.780 (0.763 ~ 0.800)
Chinese population (N=1180)	RF	0.824	0.765	0.586	0.889	0.778	0.648	0.850 (0.830 ~ 0.870)
Chinese population (N=1180)	GBM	0.796	0.726	0.551	0.873	0.746	0.616	0.813 (0.790 ~ 0.835)
Chinese population (N=1180)	XGBoost	0.734	0.811	0.603	0.865	0.794	0.639	0.820 (0.797 ~ 0.843)
Turkish and Kazakh population (N=209)	RF	0.675	0.754	0.552	0.786	0.713	0.580	0.789 (0.734 ~ 0.833)
Turkish and Kazakh population (N=209)	GBM	0.687	0.681	0.491	0.773	0.671	0.549	0.735 (0.670 ~ 0.790)
Turkish and Kazakh population (N=209)	XGBoost	0.689	0.743	0.551	0.791	0.695	0.557	0.755 (0.700 ~ 0.811)
American and Canadian population (N=311)	RF	0.710	0.484	0.397	0.767	0.600	0.494	0.607 (0.545 ~ 0.668)
American and Canadian population (N=311)	GBM	0.688	0.510	0.406	0.759	0.580	0.479	0.574 (0.514 ~ 0.630)
American and Canadian population (N=311)	XGBoost	0.538	0.627	0.405	0.728	0.618	0.443	0.551 (0.491 ~ 0.607)

Note: Macro averages were used to calculate all metrics. The additional candidate indicators identified, beyond those identified simultaneously by seven methods, were: *Porphyromonas*, *Lachnospiraceae ND3007 group*, *Lachnospiraceae UCG-004*, *Eubacterium xylanophilum group*, *Eubacterium siraeum group*, *Intestinibacter*, *Bifidobacterium*, *Bilophila*, *Turicibacter*, *Dorea*, *Eggerthella*, *Erysipelotrichaceae UCG-003*, *CAG-56*, *Lachnospiraceae FCS020 group*, *Lachnospiraceae NK4A136 group*, *Ruminococcus gauvreauii group*, *Monoglobus*, *Colidextribacter*, *Terrisporobacter*, *Tyzzerella*, *Eubacterium ruminantium group*, *Veillonella*, *Corynebacterium*, *Agathobacter*, *Halomonas*, *Haemophilus*, *Raoultibacter*, *Hungatella*, *Lachnospiraceae UCG-010*, *Family XIII UCG-001*, *Ruminococcus gnavus group*, *Weissella*, *Rhodococcus*, *Asteroleplasma*, *Family XIII AD3011 group*, *Rothia*, *Leuconostoc*, *Mitsuokella*, *Sphingomonas*, *CAG-873*, *Candidatus Soleaferrea*, *Finegoldia*, and *Lachnospiraceae NC2004 group*. PPV: Positive predictive value; NPV: Negative predictive value; AUC: Area under the receiver operating characteristic curve; CI: Confidence interval; RF: Random Forest; GBM: Gradient Boosting Machine; XGBoost: Extreme Gradient Boosting.

Supplementary Table 6. Performance of discriminative models constructed by candidate indicators identified in different subgroups on the test set

Groups	Model	Sensitivity	Specificity	PPV	NPV	Accuracy	F1 score	AUC(95% CI)
Chinese population (N=1180)	RF	0.781	0.772	0.588	0.865	0.759	0.624	0.830 (0.808 ~ 0.850)
Chinese population (N=1180)	GBM	0.719	0.769	0.565	0.84	0.747	0.596	0.775 (0.752 ~ 0.801)
Chinese population (N=1180)	XGBoost	0.778	0.727	0.565	0.849	0.714	0.592	0.794 (0.773 ~ 0.818)
Turkish and Kazakh population (N=209)	RF	0.763	0.673	0.536	0.817	0.707	0.611	0.794 (0.741 ~ 0.833)
Turkish and Kazakh population (N=209)	GBM	0.791	0.637	0.520	0.807	0.697	0.623	0.777 (0.722 ~ 0.822)
Turkish and Kazakh population (N=209)	XGBoost	0.822	0.587	0.488	0.818	0.675	0.605	0.752 (0.696 ~ 0.799)
American and Canadian population (N=311)	RF	0.756	0.622	0.458	0.78	0.644	0.529	0.724 (0.679 ~ 0.768)
American and Canadian population (N=311)	GBM	0.652	0.672	0.450	0.747	0.626	0.473	0.696 (0.647 ~ 0.744)
American and Canadian population (N=311)	XGBoost	0.624	0.651	0.449	0.739	0.656	0.502	0.675 (0.625 ~ 0.728)

Note: Macro averages were used to calculate all metrics. PPV: Positive predictive value; NPV: Negative predictive value; AUC: Area under the receiver operating characteristic curve; CI: Confidence interval; RF: Random Forest; GBM: Gradient Boosting Machine; XGBoost: Extreme Gradient Boosting.

Supplementary Table 7. Ranking of variable importance of different models in the Chinese population (candidate indicators identified from this population)

Variable	RF	GBM	XGBoost	Average value
Akkermansia	1.00	6.00	1.00	2.67
Faecalibacterium	3.00	1.00	5.00	3.00
Parabacteroides	5.00	8.00	2.00	5.00
Butyricicoccus	7.00	5.00	3.00	5.00
Fusicatenibacter	9.00	4.00	6.00	6.33
Bacteroides	4.00	2.00	15.00	7.00
Romboutsia	6.00	11.00	4.00	7.00
Dialister	14.00	3.00	8.00	8.33
Collinsella	8.00	14.00	7.00	9.67
Enterobacter	2.00	15.00	13.00	10.00
Roseburia	13.00	7.00	14.00	11.33
Alistipes	11.00	13.00	11.00	11.67
Flavonifractor	16.00	9.00	10.00	11.67
Parasutterella	18.00	10.00	9.00	12.33
Phascolarctobacterium	10.00	12.00	16.00	12.67
Lactobacillus	12.00	17.00	12.00	13.67
Bilophila	19.00	18.00	17.00	18.00
Erysipelatoclostridium	17.00	21.00	20.00	19.33
CAG-56	24.00	16.00	18.00	19.33
Lachnospira	22.00	20.00	19.00	20.33
Acinetobacter	15.00	25.00	22.00	20.67
Family XIII AD3011 group	23.00	23.00	21.00	22.33
Lachnospiraceae UCG-001	26.00	19.00	23.00	22.67
Eubacterium eligens group	25.00	22.00	25.00	24.00
Enterococcus	20.00	30.00	24.00	24.67
Leuconostoc	21.00	27.00	28.00	25.33

Agathobacter	27.00	26.00	27.00	26.67
Methanobrevibacter	30.00	24.00	26.00	26.67
Sellimonas	28.00	29.00	30.00	29.00
Corynebacterium	31.00	28.00	29.00	29.33
Olsenella	29.00	31.00	31.00	30.33

RF: Random Forest; GBM: Gradient Boosting Machine; XGBoost: Extreme Gradient Boosting.

Supplementary Table 8. Ranking of variable importance of different models in the Turkish and Kazakh population (candidate indicators identified from this population)

Variable	RF	GBM	XGBoost	Average value
Collinsella	1.00	1.00	1.00	1.00
Parabacteroides	2.00	2.00	2.00	2.00
Bifidobacterium	3.00	3.00	3.00	3.00
Hungatella	6.00	4.00	4.00	4.67
Flavonifractor	7.00	5.00	5.00	5.67
Senegalimassilia	5.00	7.00	6.00	6.00
Tyzzerella	8.00	8.00	8.00	8.00
Lactococcus	10.00	6.00	9.00	8.33
Methanobrevibacter	4.00	11.00	11.00	8.67
Rikenellaceae RC9 gut group	11.00	9.00	7.00	9.00
Megasphaera	9.00	10.00	10.00	9.67

RF: Random Forest; GBM: Gradient Boosting Machine; XGBoost: Extreme Gradient Boosting.

Supplementary Table 9. Ranking of variable importance of different models in the American and Canadian population (candidate indicators identified from this population)

Variable	RF	GBM	XGBoost	Average value
Actinomyces	1.00	1.00	1.00	1.00
Fusicatenibacter	2.00	2.00	2.00	2.00
Adlercreutzia	3.00	3.00	3.00	3.00
Dialister	4.00	7.00	5.00	5.33
Granulicatella	6.00	4.00	6.00	5.33
Slackia	9.00	5.00	4.00	6.00
Gastranaerophilales	5.00	8.00	7.00	6.67
Lachnospiraceae UCG-008	7.00	6.00	8.00	7.00
Faecalitalea	8.00	9.00	9.00	8.67
Lachnospiraceae UCG-010	10.00	11.00	12.00	11.00
Fusobacterium	11.00	13.00	11.00	11.67
Corynebacterium	14.00	12.00	10.00	12.00
Faecalibaculum	12.00	14.00	13.00	13.00
Senegalimassilia	15.00	10.00	14.00	13.00
Weissella	13.00	17.00	17.00	15.67
Butyrivibrio	19.00	15.00	15.00	16.33
Anaerofilum	16.00	18.00	16.00	16.67
Enterorhabdus	17.00	16.00	18.00	17.00
UCG-004	18.00	19.00	19.00	18.67
Lachnospiraceae ND3007 group	20.00	22.00	20.00	20.67
Pseudomonas	21.00	20.00	21.00	20.67
Megamonas	22.00	21.00	22.00	21.67
Alloscardovia	23.00	24.00	24.00	23.67
Fenollaria	29.00	23.00	28.00	26.67
Peptoniphilus	24.00	35.50	26.00	28.50
Staphylococcus	25.00	35.50	25.00	28.50
Bacteroides pectinophilus group	28.00	35.50	23.00	28.83
Victivallaceae	26.00	35.50	31.00	30.83

Clostridioides	30.00	35.50	29.00	31.50
Finegoldia	27.00	35.50	33.00	31.83
Muribaculum	33.00	35.50	30.00	32.83
Abiotrophia	38.00	35.50	32.00	35.17
Coriobacteriaceae UCG-003	43.00	35.50	27.00	35.17
Dysgonomonas	31.00	35.50	40.00	35.50
TM7x	32.00	35.50	40.00	35.83
Anaeroplasma	34.00	35.50	40.00	36.50
Neisseria	35.00	35.50	40.00	36.83
Enorma	36.00	35.50	40.00	37.17
Serratia	37.00	35.50	40.00	37.50
Ezakiella	39.00	35.50	40.00	38.17
Hafnia-Obesumbacterium	40.00	35.50	40.00	38.50
Succinivibrio	41.00	35.50	40.00	38.83
Comamonas	42.00	35.50	40.00	39.17
Peptoclostridium	44.00	35.50	40.00	39.83
Epulopiscium	45.00	35.50	40.00	40.17
Libanicoccus	46.00	35.50	40.00	40.50

Note: The DESeq2 method failed to identify the candidate indicator in this population, candidate indicators identified by three or more of the remaining six methods were combined as the final candidate indicators. RF: Random Forest; GBM: Gradient Boosting Machine; XGBoost: Extreme Gradient Boosting.

Supplementary Table 10. Comparison of candidate indicators identified in different subgroups with those identified in the overall population

Candidate indicators from overall population	Candidate indicators from Chinese population	Candidate indicators from Turkish and Kazakh population	Candidate indicators from American and Canadian population#
Faecalibacterium	Akkermansia*	Collinsella*	Actinomyces
Akkermansia	Faecalibacterium*	Parabacteroides*	Fusicatenibacter*
Bacteroides	Parabacteroides*	Bifidobacterium	Adlercreutzia
Parabacteroides	Butyricicoccus*	Hungatella	Dialister*
Fusicatenibacter	Fusicatenibacter*	Flavonifractor	Granulicatella
Roseburia	Bacteroides*	Senegalimassilia	Slackia
Collinsella	Romboutsia*	Tyzzerella	Gastranaerophilales
Dialister	Dialister*	Lactococcus*	Lachnospiraceae UCG-008
Romboutsia	Collinsella*	Methanobrevibacter*	Faecalitalea
Butyricicoccus	Enterobacter*	Rikenellaceae RC9 gut group	Lachnospiraceae UCG-010
Clostridium sensu stricto 1	Roseburia*	Megasphaera	Fusobacterium
Coprococcus	Alistipes*		Corynebacterium
Parasutterella	Flavonifractor		Faecalibaculum
Sutterella	Parasutterella*		Senegalimassilia
Alistipes	Phascolarctobacterium*		Weissella
Phascolarctobacterium	Lactobacillus*		Butyrivibrio
Lactobacillus	Bilophila		Anaerofilum
Enterobacter	Erysipelatoclostridium*		Enterorhabdus
Eubacterium ventriosum group	CAG-56		UCG-004
Lachnospira	Lachnospira*		Lachnospiraceae ND3007 group
Eubacterium eligens group	Acinetobacter*		Pseudomonas
UCG-003	Family XIII AD3011 group		Megamonas
Erysipelatoclostridium	Lachnospiraceae UCG-001*		Alloscardovia
Acinetobacter	Eubacterium eligens group*		Fenollaria
RF39	Enterococcus*		Peptoniphilus*
Lachnospiraceae UCG-001	Leuconostoc		Staphylococcus
Lactococcus	Agathobacter		Bacteroides pectinophilus group
Enterococcus	Methanobrevibacter*		Victivallaceae
Eisenbergiella	Sellimonas*		Clostridioides

Peptoniphilus	Corynebacterium		Finegoldia
Sellimonas	Olsenella*		Muribaculum
Methanobrevibacter			Abiotrophia
Eubacterium			Coriobacteriaceae UCG-003
Olsenella			Dysgonomonas
Oligella			TM7x
			Anaeroplasma
			Neisseria
			Enorma
			Serratia
			Ezakiella
			Hafnia-Obesumbacterium
			Succinivibrio
			Comamonas
			Peptoclostridium
			Epulopiscium
			Libanicoccus

# The DESeq2 method failed to identify the candidate indicator in this population, candidate indicators identified by three or more of the remaining six methods were combined as the final candidate indicators.

* The candidate indicator identified in this subgroup was also present in the overall population.
